# Reptile species richness associated to ecological and historical variables in Iran

**DOI:** 10.1038/s41598-020-74867-3

**Published:** 2020-10-23

**Authors:** Anooshe Kafash, Sohrab Ashrafi, Masoud Yousefi, Eskandar Rastegar-Pouyani, Mahdi Rajabizadeh, Faraham Ahmadzadeh, Marc Grünig, Loïc Pellissier

**Affiliations:** 1 Department of Environmental Sciences, Faculty of Natural Resources, University of Tehran, Karaj, Iran; 2 Institute of Terrestrial Ecosystems, ETH Zurich, Zurich, Switzerland; 3 Swiss Federal Institute for Forest, Snow and Landscape Research (WSL), Birmensdorf, Switzerland; 4 Ecology and Conservation Research Group (ECRG), Department of Environmental Sciences, Faculty of Natural Resources, University of Tehran, Karaj, Iran; 5 Department of Biology, Faculty of Science, Hakim Sabzevari University, Sabzevar, Iran; 6 Department of Computer Science, Tarbiat Modares University, Tehran, Iran; 7 Department of Biodiversity and Ecosystem Management, Environmental Sciences Research Institute, Shahid Beheshti University, Tehran, Iran

**Keywords:** Biodiversity, Biogeography

## Abstract

Spatial gradients of species richness can be shaped by the interplay between historical and ecological factors. They might interact in particularly complex ways in heterogeneous mountainous landscapes with strong climatic and geological contrasts. We mapped the distribution of 171 lizard species to investigate species richness patterns for all species (171), diurnal species (101), and nocturnal species (70) separately. We related species richness with the historical (past climate change, mountain uplifting) and ecological variables (climate, topography and vegetation). We found that assemblages in the Western Zagros Mountains, north eastern and north western parts of Central Iranian Plateau have the highest number of lizard species. Among the investigated variables, annual mean temperature explained the largest variance for all species (10%) and nocturnal species (31%). For diurnal species, temperature change velocity shows strongest explained variance in observed richness pattern (26%). Together, our results reveal that areas with annual temperature of 15–20 °C, which receive 400–600 mm precipitation and experienced moderate level of climate change since the Last Glacial Maximum (LGM) have highest number of species. Documented patterns of our study provide a baseline for understanding the potential effect of ongoing climate change on lizard diversity in Iran.

## Introduction

Exploring historical and current drivers of species richness can inform on how those gradients might be reshaped in the future^1–3^. Present environmental variables including climate, habitat heterogeneity, productivity represent important drivers of species distribution and diversity^4–7^. Diversity of present assemblages is not always in equilibrium with current climate. Thus, past climate changes may better reflect species richness^2^ or global endemism than current climate^8,9^. Beyond the Quaternary, mountain uplifting might also be associated to the formation of biodiversity gradients, shaping an association between habitat heterogeneity and species richness^10,11^. Together, it appears there is no single driver of species distribution across broad geographical range and species distributional patterns are shaped by the interplay between different historical and ecological factors^2,3,11–13^. The interplay between contemporary and historical variables in shaping biodiversity might vary between ecosystems types, biogeographic regions and taxonomic groups^2,3,11,13^.

With ca. 10,970 known species, reptiles are a highly diverse group of vertebrates^14^, but they are generally less studied compared to other vertebrate groups such as birds and mammals^5,15,16^. Reptiles are characterized by low dispersal ability and narrow ecological niches^17^. Thus, they are suitable biological models to assess the role of historical factors in shaping spatial distribution of biodiversity^18,19^. Global coarse scale distribution maps indicate that reptile richness is highest in pantropical including Central America, South America, south of Africa, Southeast Asia and Australia^20^. But some regions are less known than others, and lack high resolution distribution information. However, lizard richness is somehow different from reptile as their numbers are found to be maximum in both tropical and arid regions and reach a peak in Australia^20^. In particular, the highest proportions of threatened and Data Deficient^21^ reptiles are occurring in tropical areas^20^. Agriculture, biological resource use and urban development are the most important threats to reptile worldwide^20^.

Climate and topography are introduced as the most important contemporary determinants of reptile richness at global and regional scales^22–25^. In fact, reptile richness is highest in areas which characterized with high temperature and high topographic heterogeneity^22–25^. But the variables associated with the richness of reptile at regional scale in subtropical and desert areas were less investigated. While, reptiles have previously been the targets of richness mapping^22,26–28^, the influence of historical processes on their richness in southwest Asia was rarely quantified^29–31^. Among the historical factors, the influence of Quaternary climatic oscillations and mountains uplifting was observed on individual species, using genetic data and species distribution modelling^32–37^.

Iran is one of the biologically diverse countries in southwest Asia^38^, it is home to ca. 241 reptile species, of which ca. 71 are endemic to the country^39^. The country is a suitable place to test the effects of historical events on distribution of biodiversity. Iran’s current topography was shaped by uplifting of the different mountain ranges, which facilitated species diversification by providing new unoccupied niches^34,35,37,40^. The country also experienced several glacial periods^41^, which influenced reptile distribution^36,42,43^. Despite the fact that natural history studies have been initiated over 300 years ago in Iran^38^, the distribution and ecology of many species occupying that region remain poorly known^38,44,45^. Recent discoveries of novel lizards and snakes in Iran suggest that the biodiversity of Iran is still poorly explored and should receive further attention^46,47^. Distributions of some species like *Heremites vittatus*, *Saara loricate* and *Phrynocephalus scutellatus* were mapped using species distribution modeling^45,48,49^. Based on these studies, climate was the most important determinant of reptile distribution in Iran. Climate was also identified as a most influential variable in shaping reptile richness in the country^30^. However, there is a knowledge gap in the ecology and distribution of lizard species in Iran^44,45,49^. In this study, we mapped the species distribution of all lizards in Iran and quantified reptile richness. We associated species richness to ecological drivers of reptile distributions in Iran^30^, but considered in addition Quaternary climatic oscillations and mountains uplifting events. We had the following expectations:

1. The age of mountain uplifting have shaped topography as well as Quaternary glaciation should be associated to the present diversity of lizard species in Iran.
2. Among the ecological factors climatic variables are more influential in shaping lizard richness patterns.
3. Because nocturnal and diurnal species have different natural history, their richness is expected to be shaped by different historical and ecological variables.

## Results

### Species richness patterns

The 171 lizard species belonged to ten families and 47 genera. Family Gekkonidae and genus *Eremias* were most divers family and genus in Iran with 51 and 20 species respectively. Of 171 recognized lizard species in Iran 101 species (59.06%) were diurnal, 70 species nocturnal (40.93%) and 62 species endemic (36.25%).

**Figure 1:**
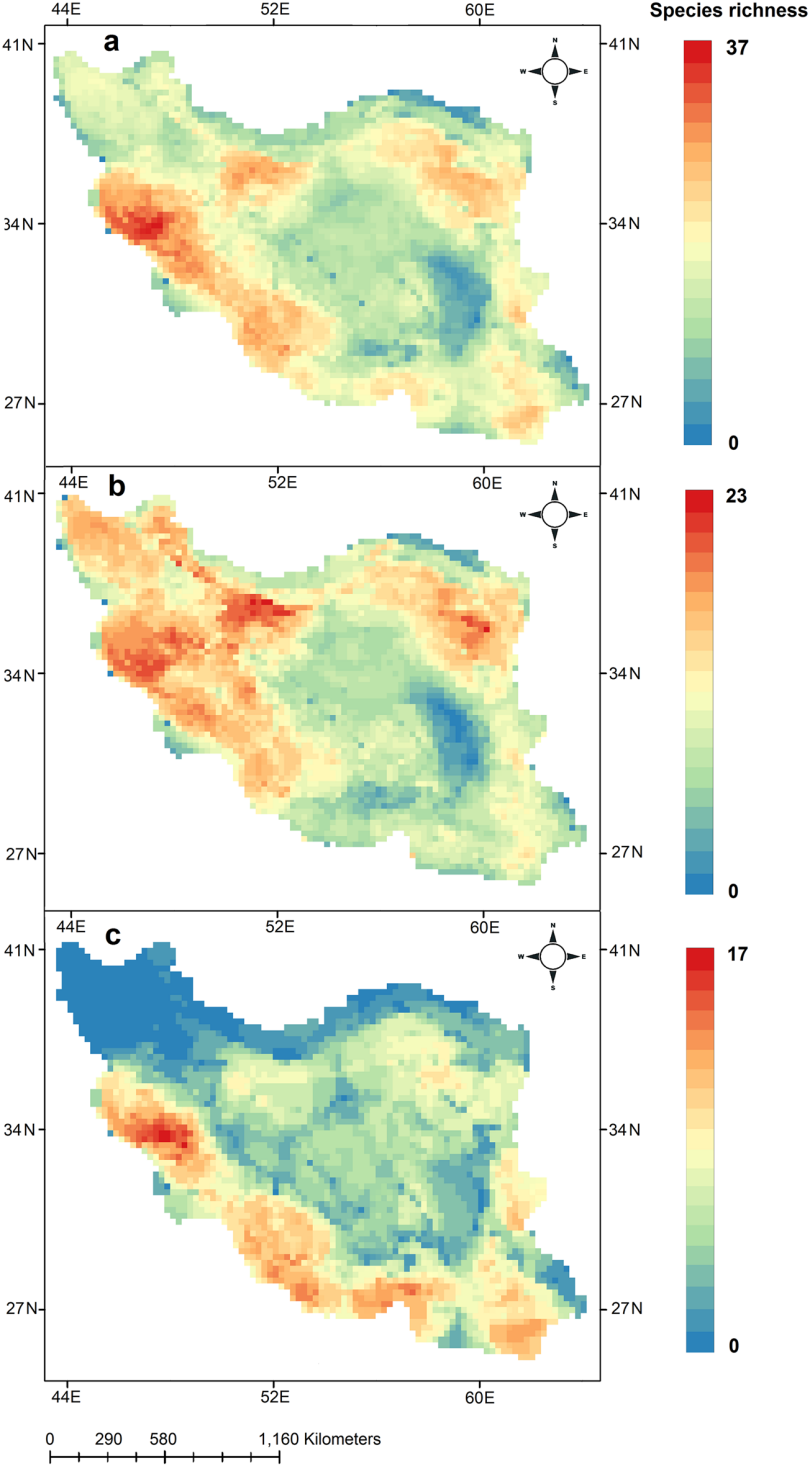
Richness map of all (**a**) diurnal (**b**) and nocturnal (**c**) lizards in Iran. All species richness range from 0 to 37, diurnal from 0 to 23 and nocturnal from 0 to 17. Maps were generated using QGIS 3.4.1 (https://www.qgis.org).

The mapping of the distribution of 171 lizard species showed that Western Zagros Mountains with 37 species, north western parts of Central Iranian Plateau with 30 species and north eastern parts with 28 species have the highest lizard richness in Iran (Fig. 1a). In contrast, northern parts of the country, Kopet-Dagh Mountains and Elburz Mountains with 2–14 species have relatively lower number of species. For diurnal species, Zagros Mountains with 22 species, north eastern and north western parts of Central Iranian Plateau with 23 species show the highest number of species (Fig. 1b). Nocturnal species hotspots occur in Western Zagros Mountains with 17 species, north of Persian Gulf and Oman Sea with 13 species, while we found that central parts of Iran with 1–6 species, Kopet-Dagh Mountains and Elburz Mountains with 0–4 species have much lower number of nocturnal species across the country (Fig. 1c). Results showed that hotspots of all lizards contain 37 species, diurnal lizard 23 and nocturnal lizards 17 species. Lut Desert contains lowest number of species for the three groups.

### Drivers of lizard richness in Iran

We found that all historical and ecological variables are significantly correlated with lizard richness (Table 1). We determined which variable explained highest proportion of variance among ecological and historical variable. Results showed that annual mean temperature explained largest variance for all species (10%) and nocturnal species (31%). For diurnal species, velocity of temperature shows strongest effect in explaining variance in observed richness pattern (26%). The distribution of lizard richness has a positive relationship with annual mean temperature and the number of species increases with higher mean temperatures for the three groups (all species, diurnal and nocturnal). Topographic heterogeneity is positively correlated with lizard species richness but NDVI was negatively correlated with richness for the three groups. Richness is negatively correlated with precipitation for the three groups. Temperature change velocity is positively associated with higher number of species for all lizards, diurnal and nocturnal lizards and maximum number of species occur in areas with moderate level of temperature change velocity.

**Table 1:** Results of generalized linear model with quasi-Poisson distribution.

Group	Predictor	Slope (linear)	z value	AIC	D2	Predictor	Slope (quadratic)	z value	AIC	D2
**All species**										
	Intercept	2.21E+00	75.74***			Intercept	2.796446	585.197***		
	**Temperature**	**1.34E−03**	27.795***	**18,740**	**0.0494**	**Temperature**	**− 8.5712**	− 18.493***	**16,830**	**0.1055**
	Temperature velocity	1.24E−03	8.614***	18,760	0.0457	Precipitation	− 2.14503	− 7.236***	18,370	0.0253
	Precipitation	− 1.59E−03	− 14.073***	18,910	0.0224	Temperature velocity	− 1.7505	− 3.887***	18,630	0.0654
	Topo-heterogeneity	5.71E−03	1.961*	18,940	0.0177	NDVI	− 0.98842	− 3.883***	18,700	0.0559
	Precipitation velocity	4.67E−04	5.635***	19,050	0.0014	Precipitation velocity	0.174253	0.581	18,830	0.0355
	NDVI	− 1.22E+00	− 21.612***	19,060	0.0002	Topo-heterogeneity	0.958962	3.633***	18,880	0.0274
	Geo	5.40E−10	1.931	19,060	0	Geo	2.833519	9.111***	19,060	0
	Full model	–	–	17,680	0.213	Full model	–	–	16,830	0.3437
**Diurnal**										
	Intercept	2.36E+00	71.152***			Intercept	2.409271	444.02***		
	**Temperature velocity**	**2.01E−03**	16.463***	**18,060**	**0.1243**	**Temperature velocity**	**− 9.40342**	− 16.718***	**17,220**	**0.2685**
	Temperature	1.11E−03	21.284***	18,080	0.1079	NDVI	− 0.30654	− 0.612	17,750	0.1715
	Precipitation velocity	1.36E−03	14.359***	18,300	0.0704	Temperature	− 3.00657	− 8.113***	18,020	0.122
	Precipitation	− 1.37E−03	− 7.857***	18,400	0.0526	Precipitation velocity	− 0.21334	− 0.573	18,220	0.0846
	NDVI	− 8.31E−01	− 13.794***	18,460	0.041	Precipitation	− 1.23191	− 4.006***	18,320	0.0656
	Topo-heterogeneity	3.58E−02	10.995***	18,630	0.0092	Topo-heterogeneity	− 0.40011	− 1.257	18,630	0.009
	Geo	1.04E−09	3.25**	18,680	0	Geo	3.325387	8.851***	18,680	0.0009
	Full model	–	–	17,240	0.2658	Full model			16,600	0.5221
**Nocturnal**										
	Intercept	− 9.98E−02	− 1.919***			Intercept	1.42242	137.94***		
	**Temperature**	**8.63E−03**	45.972***	**16,750**	**0.292**	**Temperature**	**− 7.34695**	− 11.508***	**16,530**	**0.3135**
	NDVI	− 3.13E+00	− 25.14***	18,830	0.0845	Precipitation velocity	− 16.5552	− 20.062***	17,910	0.1759
	Topo-heterogeneity	7.43E−02	15.469***	19,440	0.0245	NDVI	− 11.7424	− 10.3***	18,680	0.1
	Precipitation velocity	− 1.99E−03	− 12.669***	19,550	0.013	Topo-heterogeneity	− 0.04915	− 0.104	19,370	0.0314
	Geo	3.38E−09	7.673***	19,640	0.004	Temperature velocity	− 2.05889	− 4.303***	19,610	0.0072
	Precipitation	− 2.06E−03	− 24.405***	19,670	0.0011	Geo	0.45501	0.803	19,620	0.0067
	Temperature velocity	1.57E−03	5.727***	19,670	0.0016	Precipitation	− 5.05909	− 5.223***	19,660	0.0023
	Full model	–	–	14,930	0.4726	Full model	–	–	13,590	0.6062

The table shows linear and quadratic estimates, associated z‐ values with p‐values (asterisks), the Akaike information criterion values (AIC) and explained deviance (D2) of variables. The AIC values and the explained deviance were estimated for each predictor separately and in combination for full model. The most important variables for all lizards, diurnal lizards and nocturnal lizards were indicated in bold. Significance codes: 0 ‘***’ 0.001 ‘**’ 0.01 ‘*’ 0.05.

## Discussion

We mapped the richness of all recognized lizard species in Iran, using distribution data of 171 lizard species and documented pronounced gradients of species richness, which were different between nocturnal and diurnal species. The Western Zagros Mountains, north eastern and north western parts of Central Iranian Plateau have the highest number of species, with a peak specifically in the Zagros Mountains.

Climatic variables were identified as the important drivers of reptile distribution around the globe^5,22^. Our results supported previous effects of climate and showed that temperature was the best explaining variable for richness of all and nocturnal species, while temperature change velocity explained highest fraction of variation in diurnal species richness. Hosseinzadeh, et al.^30^ also showed that temperature is the most important predictor of reptile distribution in Iran. A positive association was frequently reported for reptile richness and temperature around the world^50^. Body temperature, which is the most important ecophysiological variable affecting the performance of reptiles, is regulated by ambient energy input^51^. Annual mean temperature is an indicator of ambient energy input^52^ and it is often used as a measure of environmental energy^53^. Thus, areas with higher temperature and consequently higher ambient energy can support more species^52,53^. This is why a positive association was found between lizard richness and temperature in all three groups. In agreement with our expectations, nocturnal and diurnal lizard richness was shaped by different contemporary and historical variables. Moreover, we found that areas which receive less precipitation have more lizard species, while in other group of reptiles, such as turtles, richness may show the opposite response and increase with precipitation^54,55^. Nocturnal lizards are richest in the tropics and deserts, and their richness decreases with latitude^56^. Nocturnal lizards, especially those of the families Gekkonidae, Sphaerodactylidae and Phyllodactylidae are more divers in the south of Iran than the rest of the country^38,39^. Annual mean temperature is increased toward southern Iran, that could be linked to the increased diversity of nocturnal lizards. In warm environments, the hot days increases the cost of diurnal activity, whereas nocturnal activity provides a shelter from these extreme conditions, for e.g. feeding and reproduction.

Quaternary climatic change was associated with distribution biodiversity in high latitude^57^. Our results show that past climate change also played important role in shaping biodiversity distribution in lower latitude as well. During the ice ages, mountains of Iran were covered by snow and ice line and snow line was much lower than what we see today^41^. So reptiles of Iran have undergone several cycles of range expansion–contraction due to the climate fluctuations which in turn shaped their current distribution^36,42,43^. We found that temperature change velocity was the most important variable in explaining variation in diurnal species richness and the second most important predictor of all lizard richness. Past climate change was not important in shaping nocturnal species richness because their richness reach a peak at south of Iran^38,39^ which was not influenced by past climatic changes^41^.

Our results agree with previous studies, which have shown that past climate is a major determinant of reptile richness^13,31^. For example, Araújo, et al.^13^ showed that past climate played an important role in shaping large-scale species richness patterns of reptiles and amphibians across Europe. In another study Ficetola et al. ^31^ quantified the importance of past climate on reptiles of the Western Palearctic. In both studies, reptile richness and endemism were highest in areas with high climate stability, low climate change velocity. Our findings showed that lizard richness was positively associated with temperature change velocity, areas with moderate climate change velocity were correlated with maximum number of species. Our finding is in line with previous studies which highlighted the role of Quaternary climatic oscillations on Iran’s biodiversity using genetic data and species distribution modeling^34,42,43^. Zagros Mountains which contain highest number of species was a refugium for several taxonomic groups during past climatic oscillations including reptiles^34,42,43,58^, amphibians^59,60^ birds^61,62^, and mammals^63–65^.

As pointed by Wines and Graham^66^, there might be strong correlation between species richness and some environmental variables but environmental variables (in our case temperature) cannot change the number of species in particular region^66^. Species richness pattern linked to processes like speciation, dispersal and extinction^66,67^. Zagros uplifting caused speciation by splitting populations of species and by providing unoccupied niches for species of the genus *Eremias*, *Mesalina*, *Timon* and *Sarra*^32,35,38,68^. Climatic changes also strongly influenced lizard distribution pattern and played important role in their isolations and dispersal in Zagros region^34,69^. There are some sky island species which remained in climatic refugia in Zagros Mountains like *Iranolacerta brandtii* and *Iranolacerta zagrosica* during past climatic oscillations^34^. In addition, Zagros acted as dispersal barrier and known as dispersal corridor for different species of lizards^38,69,70^. According to our knowledge, there is not any specific driver of extinction of lizard in the area. Thus, among the three main processes which are linked to species richness in each region, speciation and dispersal due to Zagros uplifting and past climatic fluctuations, are the main drivers of lizard richness patterns (high richness in Zagros region).

In this study, Zagros Mountains were identified as hotspot of lizard richness in Iran. This region was also identified as hotspot of biodiversity for mammals and plants in the country^7,71^. Our analyses indicate that the Zagros Mountains are a region of moderate climate stability and high topographic heterogeneity, providing a high diversity of climatic niches. Furthermore, the Zagros Mountains are located in the Irano-Anatolian biodiversity hotspot^72^, showing that there are local hotspots of biodiversity nested within regional biodiversity hotspots^7^. Zagros Mountains were also identified as an endemism area for reptiles of the Western Palearctic^31^. Beside reptiles, there are some high prioritized species for conservation such as the Luristan newt (*Neurergus kaiseri*), which was ranked as 45th among the world’s 100 top priority amphibian species^73^. This combination of high diversity and endemism makes Zagros Mountains a most valuable target for conservation of biodiversity in Iran.

## Conclusion

Altogether, our results suggest that lizard richness can be explained by current and past climate in Iran. There are studies which quantified the role of ecological factors on reptile richness in southwest Asia (for instance^29–31^), while historical drivers of reptile richness remain poorly understood^31^. For better understanding the drivers of current species distribution, we need to look at past and investigate the role of historical factors on species distribution. This study took in to account both historical and ecological factors effects on the distribution patterns of 171 lizard species in Iran. Our findings support the fact that there is no single driver for biodiversity distribution and there is always a set of ecological and historical factors shaping species richness^3,11,12,74^. Since lizard richness is strongly associated with climate we speculate that lizard diversity and distribution will be affected by future climatic changes. Documented patterns of our study provide a baseline for understanding the potential effect of ongoing climate change on lizards in Iran.

## Methods

### Study area

**Figure 2:**
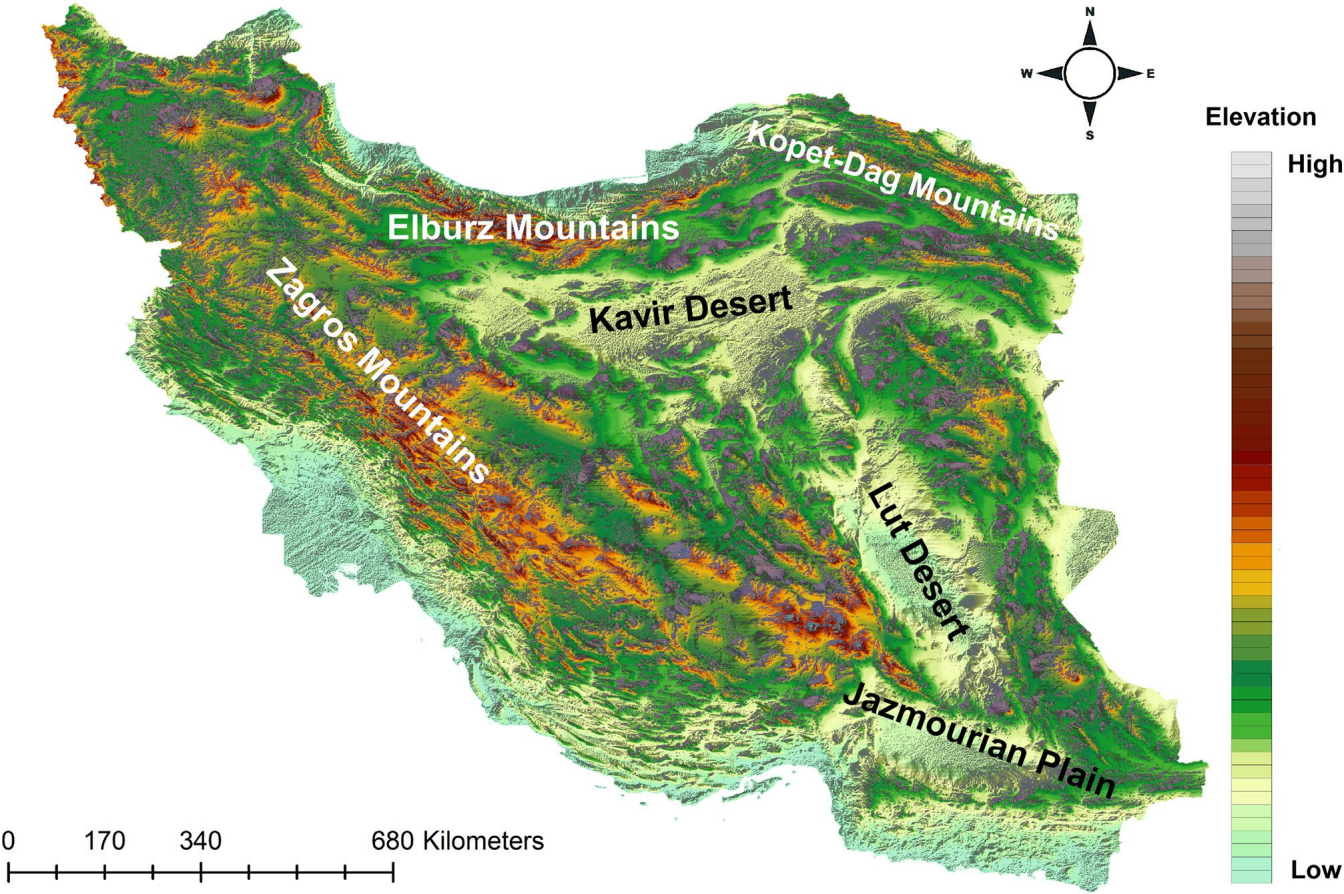
A topographic overview of Iran with its major geomorphological features. Map was generated using QGIS 3.4.1 (https://www.qgis.org).

Iran covers 164.8 million hectares, located in the Palearctic region at the crossroads of three biogeographic realms; Afrotropic, Palearctic and Indomalaya. Iran hosts over ca. 8000 plant species^75,76^ and more than 1214 vertebrate species of which many are endemic to the country^38,39,71,77^. The elevation ranges from − 26 to 5770 m and a large fraction of the country has an elevation above 1200 m. Iran’s current topography (Fig. 2) was shaped mainly via tectonic activities of Arabia–Eurasia continent collision^78^. This collision generated in around early Miocene, approximately 19 Mya^79,80^ and the last mass tectonic event in this zone occurred in late Miocene and the beginning of the Pliocene, 5 Mya, when progressive anticlockwise rotation of the Arabian Peninsula associated with the formation of the Red Sea and Gulf of Aden^81^. The collision of Arabia – Eurasia continents resulted in multi stage uplift of the Zagros Mountains, as well as the uplift of the whole plateau. The Elburz Mountains in northern Iran are stretching from west to east at the southern coast of the Caspian Sea and form the northern border of the Iranian Plateau. They are more than 1000 km long and the width varies from 30 to 130 km in different parts. The northern slopes of Elburz Mountains are covered by Hyrcanian forests. Kopet Dagh Mountains is a large mountain range (about 650 kms along) which is located in the northeast of Iran between Iran and Turkmenistan, stretching from near the Caspian Sea to the Harirud River. Zagros Mountains form the western and south-western borders of the Iranian Plateau, covering 1500 km from Lake Van in Turkish Kurdistan to south-eastern Iran. Iran has two well-known deserts, the Kavir Desert and the Lut Desert which are among the hottest areas in the world, located in Iran's central plateau. The Quaternary has been a period of global climate oscillation and several Quaternary climatic changes occurred in Iran, also on the dry Iranian highlands^82^. The most recent glaciation, termed the Riss-Würm, reached its maximum about 18,000–21,000 years ago, and subsequently replaced by Holocene Climatic Optimum (HGO), 9000–5000 years ago^83,84^. During this period, in northern and western Iran climate changed between dry and cold climatic conditions during the glaciation and moist and warm conditions during the interglacials^82–84^.

### Distribution points

Distribution records of all 171 recognised lizard species of Iran (see Appendix S1 for annotated checklist of lizards of Iran) were collected from multiple sources; (1) through opportunistic observations and long term own and other colleagues fieldworks from 2009 to 2020 (Using random field surveys different habitat types within the county were investigated (See Appendix S2 Figs. S1–S36 for examples habitats surveyed during fieldworks and observed lizard species in each habitat)), (2) published papers and books (see Appendix S3) and (3) from the Global Biodiversity Information Facility (GBIF: https://www.gbif.org/). Observed lizards were identified following Anderson^38^, Rastegar-Pouyani et al.^39^ and Narabadi et al.^85^. In total, we collected 8620 distribution points from these sources. Since our distribution records come from multiple sources, we carefully checked distribution data, removing duplicate records and distribution records lacking geographic coordinates. Reliability of all 171 lizard species’ distribution records was examined by mapping each species records separately in DIVA-GIS 7.5^86^. We also thinned distribution records of each species to 1 km reduce clustering. Finally, we used 6245 species presence records.

### Environmental variables

We selected seven variables (Table 2) related to climate: topography, the normalized difference vegetation index (NDVI), climate change velocity and geology which we expected to be important in shaping distribution of reptiles^22–24,30,32–35,38,45,48,49,87^. To quantify climate effect on reptile distribution, we used temperature and annual precipitation^13^. Current climate data was downloaded from WorldClim (www.worldclim.org). We quantified topographic heterogeneity by measuring the standard deviation of elevation values in area grid cells of 1 km from a 90 m resolution^88^. Elevation layer obtained from the Shuttle Radar Topography Mission (SRTM) elevation model^89^. The climate change velocity was calculated following Sandel et al.^9^. We used temperate and precipitation data of current climatic conditions (1970–2000) and Last Glacial Maximum (LGM; 21,000 years before present) to calculate climate change velocity as the rate of climate change in time divided by the local climate change in space. Climate change velocity is a measurement for long-time climate variability^9,90^ and it shows the direction and rate at which organisms must have moved to maintain a given climate under climate change^9,91^. In previous research, climate change velocity was identified to be strongly associated with species richness and endemism at regional and global scales^9,90,92^. LGM data were obtained from CCSM4 and MIROC 3.2 (averaged values) downloaded from WorldClim (www.worldclim.org). Temperature and precipitation change velocity were calculated in R 3.3^93^ environment using the packages raster^94^, gdistance^95^ matrixStats^96^ and SDMTools^97^. To explore the possible role of mountains uplifting on lizard richness we assembled information on uplift age of each major mountain range based on the available literatures^78,98–102^, then combined this information in a raster layer containing uplift age of the various mountain ranges in Iran. The layer with uplifting age of mountain ranges was created using QGIS 3.4.1^103^.To avoid collinearity among variables a variance inflation factor (VIF^104^) was calculated for the variables using the ‘vifstep’ function in the ‘usdm’ package^105^ in R 3.3^93^ environment and results showed that collinearity among variables is low (VIF values: annual mean temperature = 1.356, annual mean precipitation = 3.86, topographic heterogeneity = 1.614, NDVI = 3.041, temperature change velocity = 1.185, precipitation change velocity = 1.153, mountains uplifting = 1.056).

**Table 2:** List of ecological and historical predictors used to explore drivers of lizard richness.

Units	Variable (abbreviation) and references
Degrees celsius	Annual mean temperature (temperature)^106^
Millimeters	Annual precipitation (precipitation)^106^
Meters	Topographic heterogeneity (topo-heterogeneity)^89^
–	Normalized Difference Vegetation Index (NDVI)^107^
Degrees celsius	Temperature change velocity (temperature velocity)^108^
Millimeters	Precipitation change velocity (precipitation velocity)^108^
Million years	Mountain uplifting age (Geo) ^78,98–102^

### Species richness mapping

**Figure 3:**
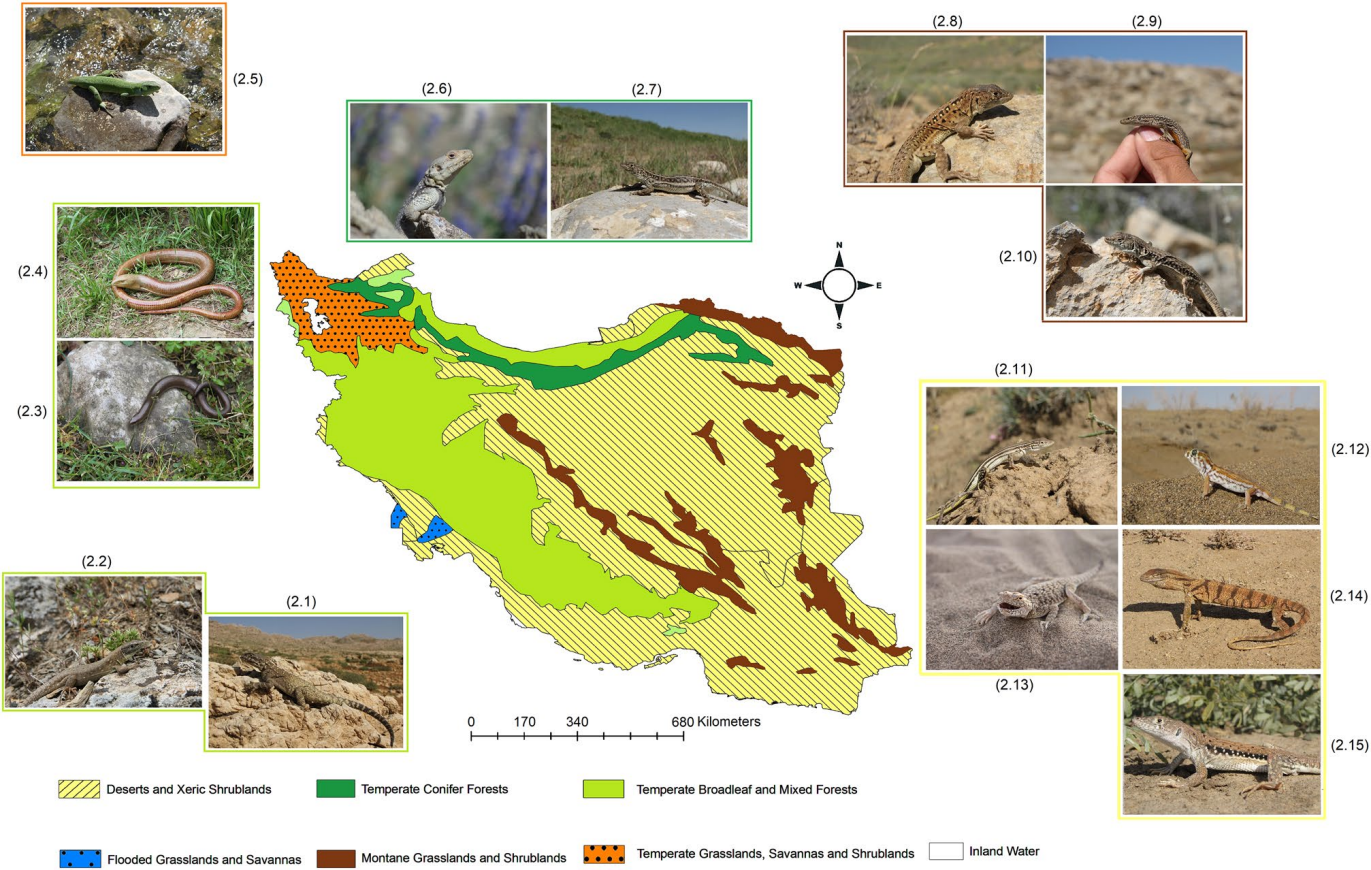
Iran’s terrestrial biomes^110^, together with representative lizards species occur in Temperate Broadleaf and Mixed Forests: 2.1 *Paralaudakia microlepis*, 2.2 *Timon kurdistanicus*, 2.3 *Anguis colchica*, 2.4 *Pseudopus apodus*; Temperate Grasslands, Savannahs, and Shrublands: 2.5 *Lacerta media*; Temperate Conifer Forests: 2.6 *Paralaudakia caucasia*, 2.7 *Eremias papenfussi*; Montane grasslands and shrublands: 2.8 *Eremias kopetdaghica*, 2.9 *Darevskia kopetdaghica,* 2.10 *Eremias isfahanica*; Deserts and Xeric Shrublands: 2.11 *Eremias fasciata*, 2.12 *Teratoscincus scincus*, 2.13 *Phrynocephalus mystaceus*, 2.14 *Varanus griseus* 2.15 *Eremias persica.* Map was generated using QGIS 3.4.1 (https://www.qgis.org).

We followed the method applied by Pellissier et al.^11^ and Albouy et al.^109^ to map species ranges and then multiply them to create lizard richness in Iran. This method uses species occurrence records, border of biomes (Fig. 3) of the study area and a climatic layer (here we used annual mean temperature an important factor in shaping reptile distribution) to create species range maps. We separated Iran into six biomes based on the World Wildlife Fund (WWF) Terrestrial Ecoregions including the following; Temperate Broadleaf and Mixed Forests, Temperate Grasslands, Savannahs and Shrublands, Temperate Conifer Forests, Montane grasslands and shrublands, and Deserts and Xeric Shrublands^110^. This approach goes through several steps to map species distribution; first, we created a convex hull polygon around all the observations within one bioregion. If there is only one observation in a bioregion, it will create a buffer around it of 50 km. Then to remove potential outliers, we quantified the 2.5th and 97.5th temperature values from the occurrence, where a species is found. We removed areas that are outside those temperature conditions. Together, we created range maps of all 171 lizard species, and we stacked the species distribution maps into species richness using raster package^94^ in R environment^93^ to create three richness maps; all lizards, diurnal lizards and nocturnal lizards.

### Statistical analyses

We fitted a generalized linear model (GLM) with quasi-Poisson distribution in order to explore the relationship between reptile richness (number of species in each raster pixel) and the different historical and ecological factors. We estimated the Akaike information criterion values (AIC) and computed the explained deviance for each predictor separately and in combination for full model using the ‘ecospat.adj.D2.glm’ function in the R‐package ‘ecospat’^111^. Nocturnal and diurnal species have different natural and evolutionary history and their distribution patterns are different in Iran^38,44^. For example, most of the nocturnal species are occur in south of Iran which characterized with high temperature and more stable climate since LGM^68^. Thus, nocturnal and diurnal lizard distribution is most likely affected by different ecological and historical variables. So, we did the analysis for all lizard, diurnal and nocturnal separately to test whether there are different drivers for diurnal and nocturnal species. Analyses were carried out in R 3.3^93^.

## Supplementary information

Supplementary Information.

## Data Availability

All data needed to evaluate the conclusions in the paper are present in the paper and/or the Supplementary Materials, or the references cited here within.
