# Supplementary material for: Reptile species richness associated to ecological and historical variables in Iran
Source: Sci Rep. 2020 Oct 23;10:18167. doi: 10.1038/s41598-020-74867-3 (PMC7584626; doi:10.1038/s41598-020-74867-3)
Supplement: Supplementary file 1 — Supplementary Information. [file 41598_2020_74867_MOESM1_ESM.pdf]

Reptile species richness associated to ecological and historical variables in Iran

A. Kafash, S. Ashrafi, M. Yousefi, E. Rastegar Pouyani, M. Rajabizadeh, F. Ahmadzadeh, M. Grünig, L. Pellissier

Appendix S1. Annotated checklist of lizards of Iran. This checklist contains 171 lizard species. Most recent checklist of lizards of Iran Safaei-Mahroo *et al.*[1] presented 148 species but since last published checklist some new endemic species discovered[2-15] and some taxonomic revisions has been made[15,16]. This checklist includes all recognized lizard until September, 2020. Based on our annotated checklist, Iran is home to 171 lizard species. These 171 lizard species belonged to ten families and 47 genera. Of 171 recognized lizard species in Iran 101 species (59.06 %) were diurnal, 70 species nocturnal (40.93 %) and 62 species endemic (36.25 %).

Number	Family	Species	Diurnal	Nocturnal	Endemic	Authors
1	Trogonophidae	*Diplometopon zarudnyi*	❈			Nikolski, 1907
2	Agamidae	*Calotes versicolor*	❈			(de Filippi, 1843)
3		*Laudakia melanura*	❈			(Eichwald, 1831)
4		*Laudakia nupta*	❈			(Nikolski, 1896)
5		*Paralaudakia caucasia*	❈			(Blanford, 1874)
6		*Paralaudakia erythrogaster*	❈			Nikolski, 1907
7		*Paralaudakia microlepis*	❈			(de Filippi, 1843)
8		*Phrynocephalus ahvazicus*	❈		❈	Melnikov, Melnikov, Nazarov, Rajabizadeh, Al-Johany, Amr & Ananjeva 2014
9		*Phrynocephalus ananjevae*	❈		❈	Melnikov, Melnikov, Nazarov, Rajabizadeh, 2013
10		*Phrynocephalus arabicus*	❈			Anderson 1894
11		*Phrynocephalus helioscopus*	❈			(Pallas, 1771)
12		*Phrynocephalus horvathi*	❈			Méhely, 1894

13		*Phrynocephalus scutellatus*	❈			(Olivier, 1807)
14		*Phrynocephalus maculatus*	❈			Anderson, 1872
15		*Phrynocephalus mystaceus*	❈			(Pallas, 1776)
16		*Phrynocephalus ornatus*	❈			Boulenger, 1887
17		*Phrynocephalus persicus*	❈			de Filippi, 1863
18		*Phrynocephalus raddei*	❈			Boettger 1888
19		*Phrynocephalus lutensis*	❈		❈	Kamali and Anderson, 2015
20		*Saara asmussi*	❈			(Strauch, 1863)
21		*Saara loricata*	❈			(Blanford, 1874)
22		*Trapelus agilis*	❈			(Olivier, 1804)
23		*Traplelus persicus*	❈			(Blanford, 1881)
24		*Trapelus ruderatus*	❈			(Olivier, 1804)
25		*Trapelus sanguinoletus*	❈			(Pallas, 1814)
26		*Uromastyx aegyptia*	❈			(Forskal, 1775)
27	Anguidae	*Anguis colchica*	❈			(Nordmann, 1840)
28		*Pseudopus apodus*	❈			(Pallas, 1775)
29	Eublepharidae	*Eublepharis turcmenicus*		❈		Darevsky, 1977
30		*Eublepharis macularius*		❈		(Blyth, 1854)
31		*Eublepharis angramainyu*		❈		Anderson & Leviton, 1966
32	Gekkonidae	*Agamura persica*		❈		(Duméril, 1856)
33		*Agamura cruralis*		❈		Blanford, 1874
34		*Agamura kermanensis*		❈	❈	Hosseiian Yousefkhani, Aliabadin, Rastegar-Pouyani, Daevish, Shafiei, Sehhatisabet, 2018
35		*Bunopus tuberculatus*		❈		Blanford, 1874
36		*Bunopus crassicauda*		❈	❈	Nikolsky, 1907
37		*Crossobamon eversmanni*		❈		(Wiegmann, 1834)
38		*Cyrtopodion agamuroides*		❈		(Nikolsky, 1900)
39		*Cyrtopodion brevipes*		❈		(Blanford, 1874)
40		*Cyrtopodion gastrophole*		❈	❈	(Werner, 1917)

41	*Cyrtopodion golubevi*	❈	❈	Nazarov, Ananjeva & Rajabizadeh, 2009
42	*Cyrtopodion hormozganum*	❈	❈	Nazarov, Bondarenko & Rajabizadeh, 2012
43	*Cyrtopodion kachhensis*	❈		(Stoliczka, 1872)
44	*Cyrtopodion kiabii*	❈	❈	Ahmadzadeh, Flecks, Torki Bohme, 2011
45	*Cyrtopodion kirmanense*	❈	❈	(Nikolsky, 1900)
46	*Cyrtopodion persepolense*	❈	❈	Nazarov, Ananjeva & Rajabizadeh, 2009
47	*Cyrtopodion scabrum*	❈		(Heyden, 1827)
48	*Cyrtopodion sistanensis*	❈	❈	Nazarov & Rajabizadeh, 2007
49	*Hemidactylus flaviviridis*	❈		Rüppell, 1835
50	*Hemidactylus persicus*	❈		Anderson, 1872
51	*Hemidactylus robustus*	❈		Heyden, 1827
52	*Hemidactylus romeshkanicus*	❈	❈	Torki, 2011
53	*Hemidactylus achaemenidicus*	❈	❈	Torki, 2020
54	*Hemidactylus pseudoromeshkanicus*	❈	❈	Torki, 2020
55	*Hemidactylus sassanidianus*	❈	❈	Torki, 2020
56	*Mediodactylus aspratilis*	❈	❈	(Anderson, 1973)
57	*Mediodactylus heterocercum*	❈		(Blanford, 1874)
58	*Mediodactylus heterophollis*	❈		(Minton, Anderson, Anderson, 1970)
59	*Mediodactylus ilamensis*	❈	❈	(Fathinia, Karamiani, Darvishnia, Heidari & Rastegar-Pouyani, 2011)
60	*Mediodactylus russowii*	❈		(Strauch, 1887)
61	*Mediodactylus sagittiferum*	❈	❈	(Nikolsky, 1900)
62	*Mediodactylus spinicauda*	❈		(Strauch, 1887)
63	*Mediodactylus stevenandersoni*	❈	❈	(Torki, 2011)
64	*Microgecko helenae*	❈		Nikolsky, 1907
65	*Microgecko latifi*	❈	❈	(Leviton & Anderson, 1972)

66		*Microgecko persicus*		❈		(Nikolsky, 1903)
67		*Microgecko varaviensis*		❈	❈	Gholamifard, Rastegar-Pouyani & Rastegar-Pouyani, 2019
68		*Pseudoceramodactylus khobarensis*		❈		Haas, 1957
69		*Rhinogecko misonnei*		❈		de Witte, 1973
70		*Stenodactylus affinis*		❈		(Murray, 1884)
71		*Trigonodactylus arabicus*		❈		(Haas, 1957)
72		*Stenodactylus doriae*		❈		(Blanford, 1874)
73		*Tenuidactylus caspium*		❈		(Eichwald, 1831)
74		*Tenuidactylus longipes*		❈		(Nikolsky, 1896)
75		*Tenuidactylus turcmenicus*		❈		(Szczerbak, 1978)
76		*Tropiocolotes naybandensis*		❈	❈	Krause, Ahmadzadeh, Moazeni, Wagner & Wilms 2013
77		*Tropiocolotes hormozganensis*		❈	❈	Rajabizadeh, Faizi, Anderson, Zarrintab & Nazarov 2018
78		*Microgecko chabaharensis*		❈	❈	Gholamifard, Rastegar-Pouyani, Rastegar-Pouyani, Khosravani, Yousefkhani & Oraei 2015
79		*Parsigecko ziaiei*		❈	❈	Safaei- Mahroo, Ghaffari & Anderson 2016
80		*Trigonodactylus persicus*		❈	❈	Nazarov, Melnikov, Rajabizadeh and Poyarkov, 2018
81		*Lakigecko aaronbaueri*		❈	❈	Torki, 2020
82		*Microgecko laki*		❈	❈	Torki, 2020
83	Lacertidae	*Acanthodactylus blanfordii*	❈			Boulenger, 1918
84		*Acanthodactylus boskianus*	❈			(Daudin , 1802)
85		*Acanthodactylus cantoris*	❈			Gnther, 1864
86		*Acanthodactylus grandis*	❈			Boulenger, 1909
87		*Acanthodactylus khamirensis*	❈		❈	Heidari, Rastegar-Pouyani, Rastegar-Pouyani & Rajabizadeh 2013
88		*Acanthodactylus micropholis*	❈			Blanford, 1874
89		*Acanthodactylus nilsoni*	❈		❈	Rastegar-Pouyani, 1998

90	*Acanthodactylus schmidti*	❈		Haas, 1957
91	*Apathya cappadocica*	❈		(Werner, 1902)
92	*Apathya yassujica*	❈	❈	(Nilson, Rastegar-Pouyani, Rastegar-Pouyani & Andern 2003)
93	*Darevskia caspica*	❈	❈	Ahmadzadeh, Flecks, Carretero, Mozaffari, Bohme, Harris, Freitas & Rodder 2013
94	*Darevskia chlorogaster*	❈		(Boulenger, 1908)
95	*Darevskia defilippii*	❈	❈	(Camerano, 1877)
96	*Darevskia kamii*	❈	❈	Ahmadzadeh, Flecks, Carretero, Mozaffari, Bohme, Harris, Freitas & Rodder 2013
97	*Darevskia kopetdaghica*	❈		Ahmadzadeh, Flecks, Carretero, Mozaffari, Bohme, Harris, Freitas & Rodder 2013
98	*Darevskia praticola*	❈		(Eversmann, 1834)
99	*Darevskia raddei*	❈		(Boettger, 1892)
100	*Darevskia schaekeli*	❈	❈	Ahmadzadeh, Flecks, Carretero, Mozaffari, Bohme, Harris, Freitas & Rodder 2013
101	*Darevskia steineri*	❈	❈	(Eiselt, 1995)
102	*Darevskia valentini*	❈		(Boettger, 1892)
103	*Eremias acutirostris*	❈		(Boulenger, 1887)
104	*Eremias andersoni*	❈	❈	Darevsky & Szczerbak, 1978
105	*Eremias arguta*	❈		(Pallas, 1773)
106	*Eremias fasciata*	❈		Blanford, 1874
107	*Eremias grammica*	❈		(Lichtenstein, 1823)
108	*Eremias intermedia*	❈		(Strauch, 1876)
109	*Eremias isfahanica*	❈	❈	Rastegar-Pouyani, Hosseinian, Rafiee, Kami, Rajabizadeh & Wink 2016
110	*Eremias kavirensis*	❈	❈	Mozaffari & Parham 2007
111	*Eremias kopetdaghica*	❈		Szczerbak, 1972

112		*Eremias lalezharica*	❉		❉	Moravec, 1994
113		*Eremias lineolata*	❉			(Nikolsky, 1896)
114		*Eremias montana*	❉		❉	Rastegar-Pouyani & Rastegar-Pouyani 2001
115		*Eremias nigrocellata*	❉			Nikolsky, 1896
116		*Eremias papenfussi*	❉		❉	Mozaffari, Ahmadzadeh & Parham, 2011
117		*Eremias persica*	❉			Blanford, 1874
118		*Eremias pleskei*	❉			Bedriaga, 1905
119		*Eremias strauchi*	❉			Kessler, 1878
120		*Eremias suphani*	❉			Basoglu & Hellmich, 1968
121		*Eremias fahimii*	❉		❉	Mozaffari, Ahmadzadeh, Saberi-Pirooz, 2020
122		*Eremias velox*	❉			(Pallas, 1771)
123		*Iranolacerta brandtii*	❉			(de Filippi, 1863)
124		*Iranolacerta zagrosica*	❉		❉	(Rastegar-Pouyani & Nikson 1998)
125		*Lacerta media*	❉			Lantz & Cyrén, 1920
126		*Lacerta strigata*	❉			Eichwald, 1831
127		*Mesalina brevirostris*	❉			Blanford, 1874
128		*Mesalina watsonana*	❉			(Stoliczka, 1872)
129		*Ophisops elegans*	❉			Ménétriés, 1832
130		*Timon kurdistanicus*	❉			Blanford, 1874
131		*Timon princeps*	❉		❉	(Blanford, 1874)
132	Phyllodactylidae	*Asaccus andersoni*		❉	❉	Torki, Fathinia, Rostami, Gharzi & Nazari-Serenjeh 2011
133		*Asaccus elisae*		❉		(Werner, 1895)
134		*Asaccus granularis*		❉	❉	Torki, 2010
135		*Asaccus griseonotus*		❉		Dixon & Anderson, 1973
136		*Asaccus iranicus*		❉	❉	Torki, Ahmadzadeh, Igaz, Avci & Kumlutas 2011

137		*Asaccus kermanshahensis*		❈	❈	Rastegar-Pouyani, 1996
138		*Asaccus kurdistanensis*		❈	❈	Rastegar-Pouyani, Nikson & Faizi 2006
139		*Asaccus nasrullahi*		❈	❈	Werner, 2006
140		*Asaccus tangestanensis*		❈	❈	Torki, Ahmadzadeh, Igaz, Avci & Kumlutas 2011
141		*Asaccus zagrosicus*		❈	❈	Torki, Ahmadzadeh, Igaz, Avci & Kumlutas 2011
142	Scincidae	*Ablepharus bivittatus*	❈			(Ménétriés, 1832)
143		*Ablepharus chernovi*	❈			Darevsky, 1953
144		*Ablepharus pannonicus*	❈			(Fitzinger, 1824)
145		*Ablepharus grayanus*	❈			(Stoliczka 1872)
146		*Heremites auratus*	❈			(Linnaeus, 1758)
147		*Heremites vittata*	❈			(Olivier, 1804)
148		*Heremites septemtaeniata*	❈			(Reuss, 1834)
149		*Chalcides ocellatus*	❈			(Forskal 1775)
150		*Eumeces schneideri*	❈			(Daudin, 1802)
151		*Eumeces persicus*	❈		❈	Faizi, Rastegrar-Pouyani, Rastegrar-Pouyani, Nazarov, Heidari, Zangi, Orlova & Poyarkov 2017
152		*Eumeces blythianus*	❈			(Anderson 1871)
153		*Eurylepis taeniolatus*	❈			Blyth, 1854
154		*Ophiomorus blanfordi*	❈			Boulenger, 1887
155		*Ophiomorus brevipes*	❈			(Blanford, 1874)
156		*Ophiomorus maranjabensis*	❈		❈	Kazemi, Farhadi Qomi, Kami & Anderson, 2011
157		*Ophiomorus nuchalis*	❈		❈	Nilson & Andrén, 1978
158		*Ophiomorus persicus*	❈		❈	(Steindachner, 1867)
159		*Ophiomorus streeti*	❈		❈	Anderson & Leviton, 1966
160		*Ophiomorus tridactylus*	❈			(Blyth, 1853)
161		*Scincus mitranus*	❈			Anderson, 1871

162		*Scincus scincus*	❉			(Linnaeus, 1758)
163	Sphaerodactylidae	*Pristurus rupestris*		❉		Blanford, 1874
164		*Teratoscincus bedriagai*		❉		Nikolsky, 1899
165		*Teratoscincus scincus*		❉		(Schlegel, 1858)
166		*Teratoscincus microlepis*		❉		Nikolsky, 1899
167		*Teratoscincus sistanense*		❉	❉	Akbarpour, Shafiei, Sehhatisabet & Damadi 2017
168		*Teratoscincus mesriensis*		❉	❉	Nazarov, Rajabizadeh, Poyarkov, Ananjeva, Melnikov & Rastegrar-Pouyani 2017
169	Varanidae	*Varanus bengalensis*	❉			(Daudin, 1802)
170		*Varanus griseus*	❉			(Daudin, 1803)
171		*Varanus nesterovi*	❉		❉	Bohme, Ehrlich, Milto, Orlov & Scholz 2015

References Appendix S1.

1. Safaei-Mahroo, B. et al. The herpetofauna of Iran: checklist of taxonomy, distribution and conservation status. *Asian Herpetol. Res.* **6**, 257–290 (2015).
2. Akbarpour, M. *et al.* A new species of frog-eyed gecko, genus *Teratoscincus Strauch*, 1863 (Squamata: Sphaerodactylidae), from southeastern Iran. *Zool. Middle East.* **63**, 296-302 (2017).
3. Faizi, H. et al. A new species of *Eumeces* Wiegmann 1834 (Sauria: Scincidae) from Iran. *Zootaxa* **4320**, 289-304 (2017).
4. Gholamifard, A., Rastegar-Pouyani, N. & Rastegar-Pouyani, E. A new species of the genus Microgecko Nikolsky, 1907 (Sauria: Gekkonidae) from the southern Zagros Mountains, Iran. *Zootaxa* **4648**: 435–454 (2019).
5. Gholamifard, A. et al. A new species of the genus Microgecko Nikolsky, 1907 (Sauria: Gekkonidae) from southern Iran. *Zootaxa* **4093**: 026–040 (2016).
6. Hosseinian Yousefkhani et al. Description of a new species of the genus *Agamura* Blanford, 1874 (Squamata: Gekkonidae) from southern Iran. *Zootaxa* **4457**, 325–331 (2018).
7. Kamali, A. & Anderson S.C. A new Iranian *Phrynocephalus* (Reptilia: Squamata: Agamidae) from the hottest place on earth and a key to the genus *Phrynocephalus* in southwestern Asia and Arabia. *Zootaxa* **3904**, 249-260 (2015).
8. Mozaffari, O., Ahmadzadeh, F., Saberi-Pirooz R. Fahimi's racerunner, a new species of the genus *Eremias* Fitzinger, 1834 (Sauria: Lacertidae) from Iran. *Zootaxa* **4768**, 565-578 (2020).
9. Nazarov, R.A., Radjabizadeh, M., Poyarkov, Jr., NA., Ananjeva, N.B., Melnikov, D.A., Rastegar Pouyani, E. 2017. A new species of Frog-eyed gecko, Genus *Teratoscincus* Strauch, 1863 (Squamata: Sauria: Sphaerodactylidae), from Central Iran. *Russ. J. Herpetol.* **24**, 291-310.
10. Rastegar-Pouyani, E. et al. A new species of the genus *Eremias* Fitzinger, 1834 (Squamata: Lacertidae) from Central Iran, supported by mtDNA sequences and morphology. *Zootaxa* **4132**, 207-220 (2016).
11. Safaei-Mahroo, B. et al. A new genus and species of gekkonid lizard (Squamata: Gekkota: Gekkonidae) from Hormozgan Province with a revised key to gekkonid genera of Iran. *Zootaxa* **4109**, 428–444 (2016).
12. Torki F. A new gecko genus from Zagros Mountains, Iran. *Amphib. reptile conserv* **14**, 55–62 (2020).

13. Torki F. Three new species of *Hemidactylus* Oken, 1817 (Squamata, Gekkonidae) from Iran. *Amphib. reptile conserv* **13**, 239–258 (2019).
14. Torki, F. A new species of dwarf gecko of the genus Microgecko (Squamata: Gekkonidae) from Iran. *Sauria* **42**, 41-54 (2020).
15. Hosseinian Yousefkhani, SS., Aliabadian, M., Rastegar-Pouyani, E. & Darvish. J. Taxonomic revision of the spider geckos of the genus *Agamura* senso lato Blanford, 1874 (Sauria: Gekkonidae) in the Iranian Plateau. *Herpetol. J.* **29**: 1-12 (2019).
16. Rajabizadeh, M., Faizi, H., Anderson, S.C., Zarrintab, M. & Nazarov, R. Taxonomic status of *Tropiocolotes* cf. *steudneri* with a description of a new species of *Tropiocolotes* (Reptilia: Squamata: Gekkonidae) in southern Iran. *Zootaxa* **4388**, 283- 291 (2018).

Appendix S2 (Figures S1-S36). Examples habitats surveyed during fieldworks and observed lizard species in each habitat. Photos by Masoud Yousefi.

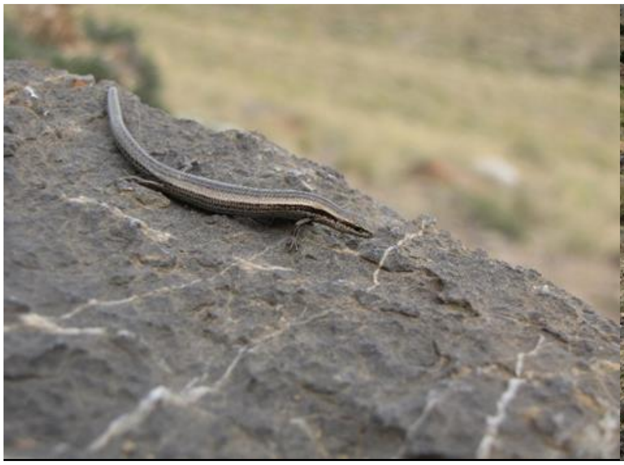

Figure S1. *Ablepharus pannonicus*.

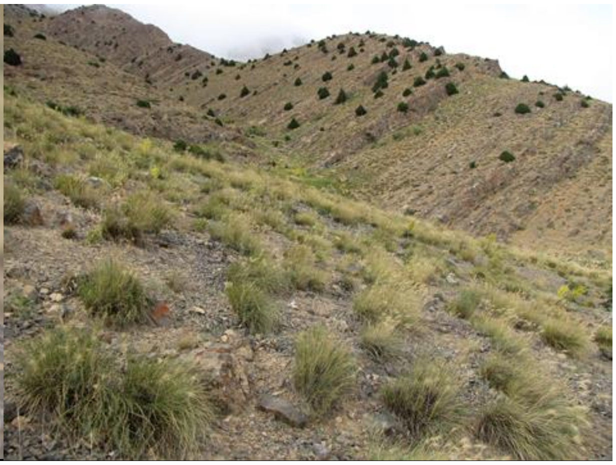

Figure S2. Habitat of *Ablepharus pannonicus*, Tehran province.

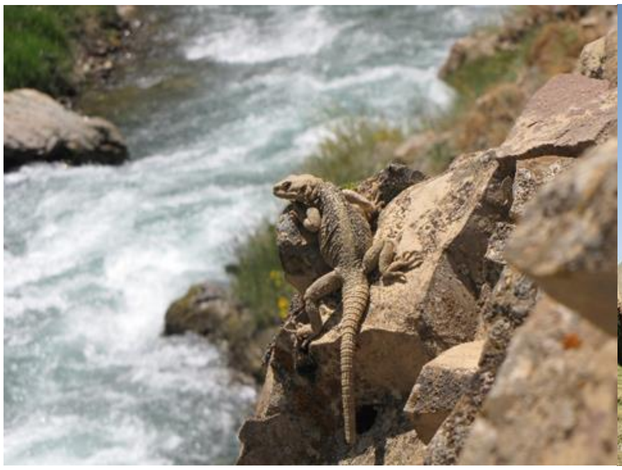

Figure S3. *Paralaudakia caucasia*.

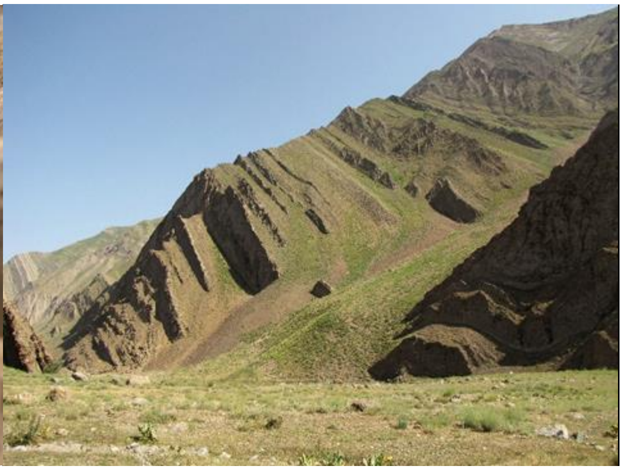

Figure S4. Habitat of *Paralaudakia caucasia*, Elburz province.

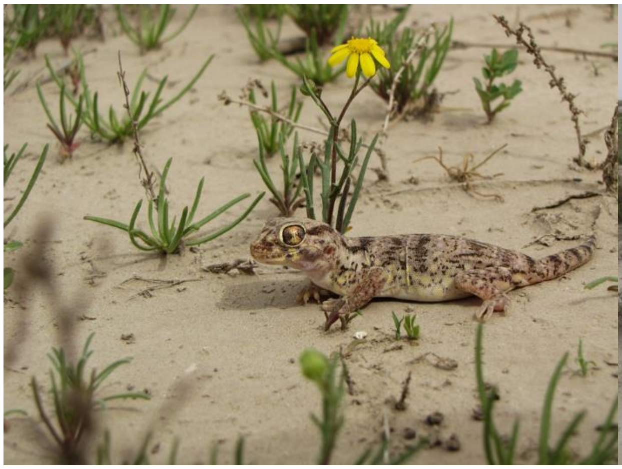

Figure S5. *Stenodactylus affinis*.

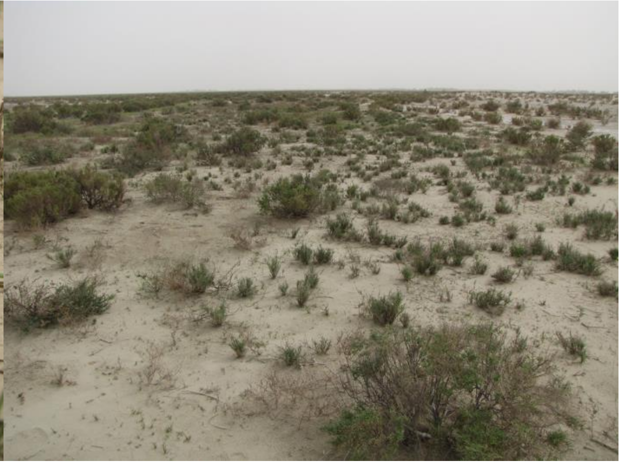

Figure S6. Habitat of *Stenodactylus affinis*, Hurmozgan province.

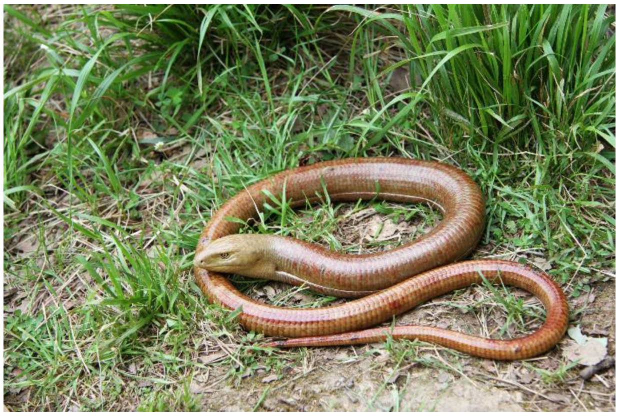

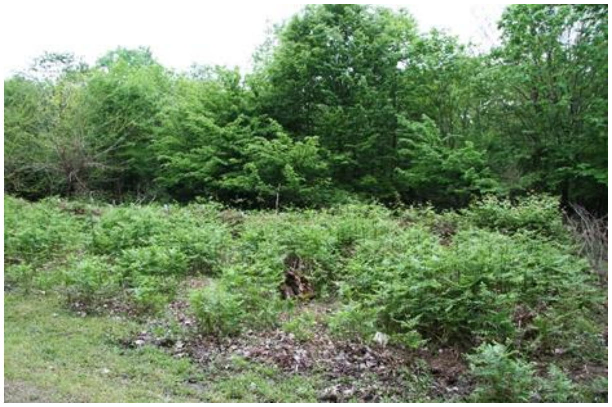

Figure S7. *Pseudopus apodus*. Figure S8. Habitat of *Pseudopus apodus*, Mazandaran province.

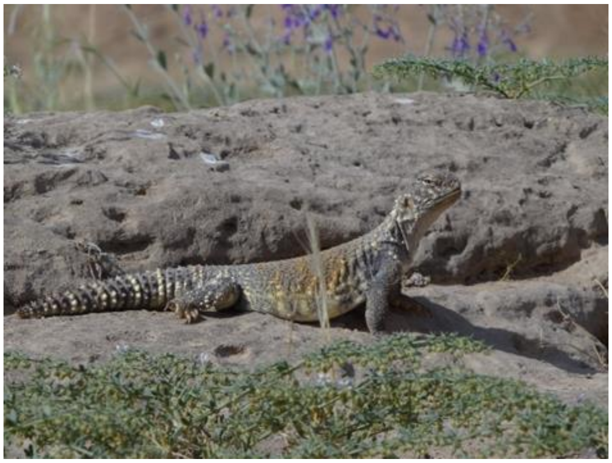

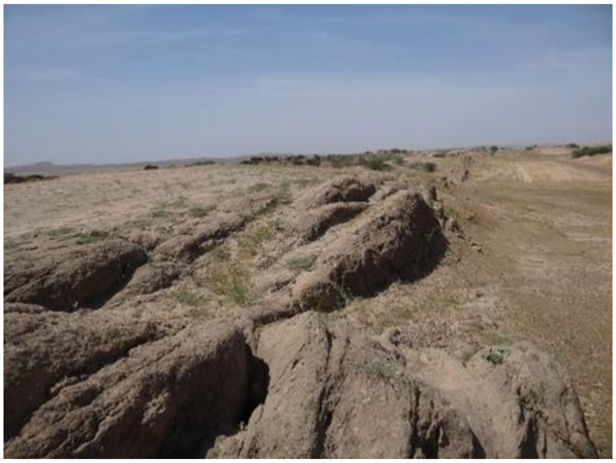

Figure S9. *Saara loricata*. Figure S10. Habitat of *Saara loricata*, Khuzestan province.

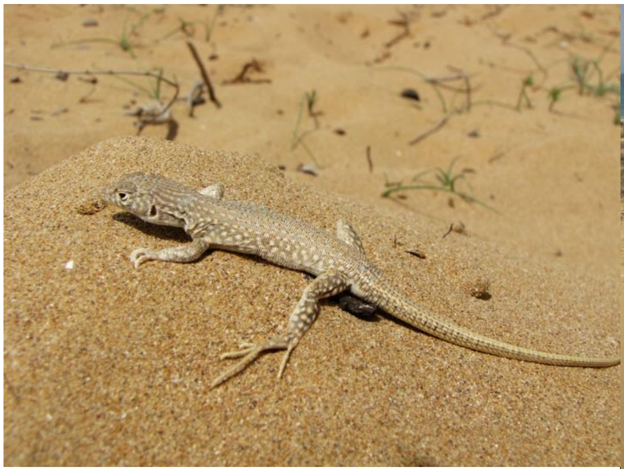

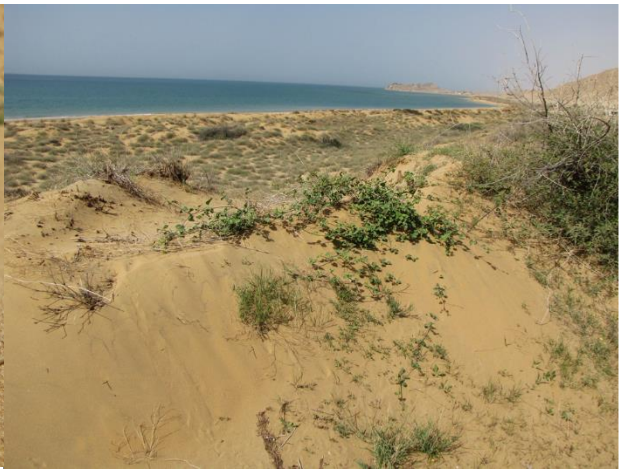

Figure S11. *Acanthodactylus khamirensis*. Figure S12. Habitat of *Acanthodactylus khamirensis*, Hurmozgan province.

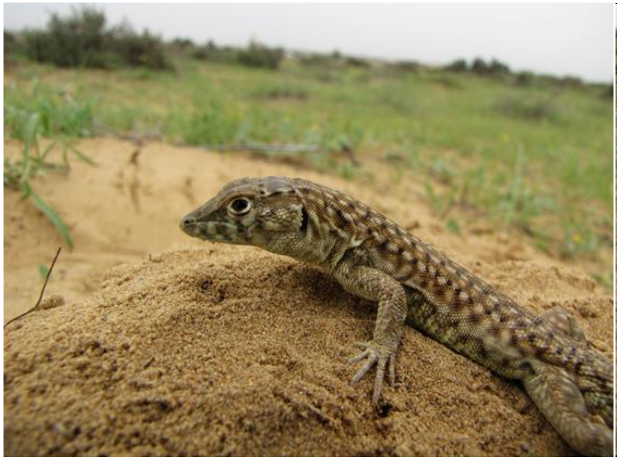
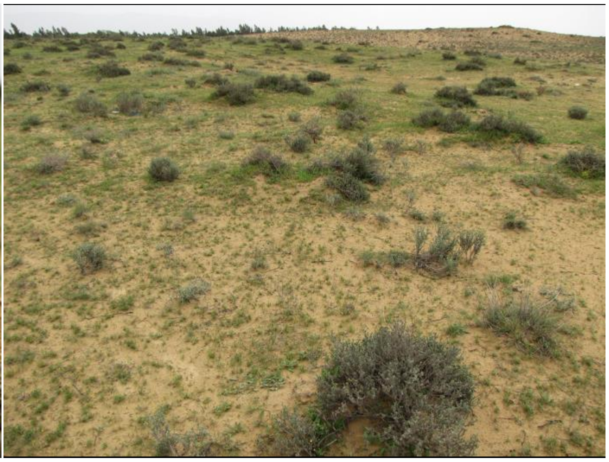

Figure S13. *Acanthodactylus grandis*. Figure S14. Habitat of *Acanthodactylus grandis*, Bushehr province.

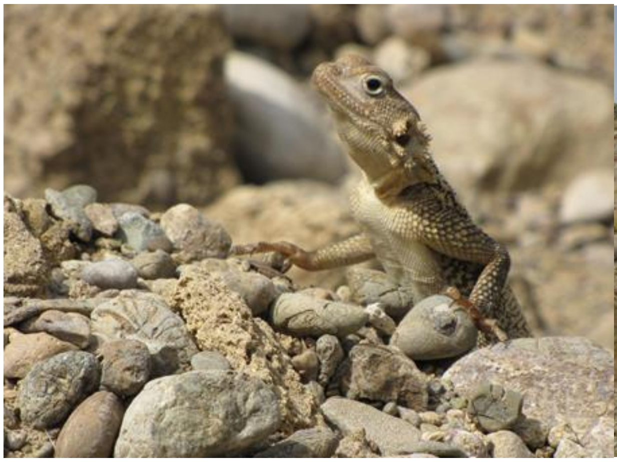
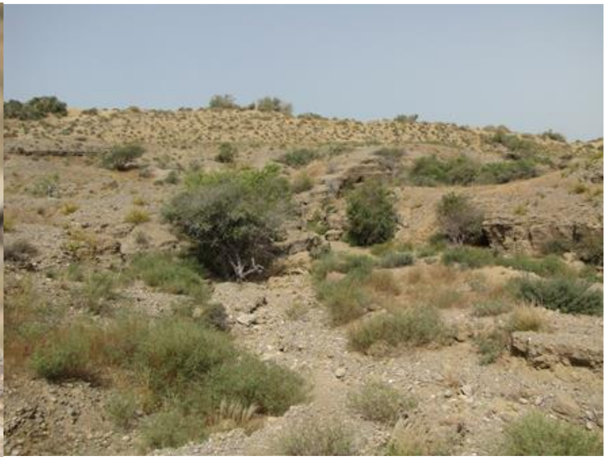

Figure S15. *Laudakia nupta*. Figure S16. Habitat of *Laudakia nupta*, Sistan and Baluchestan province.

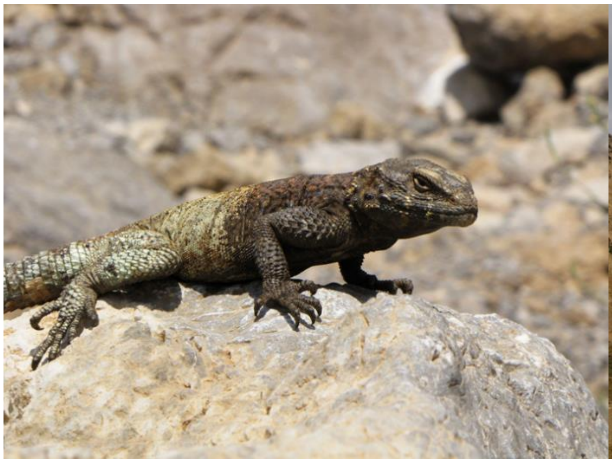
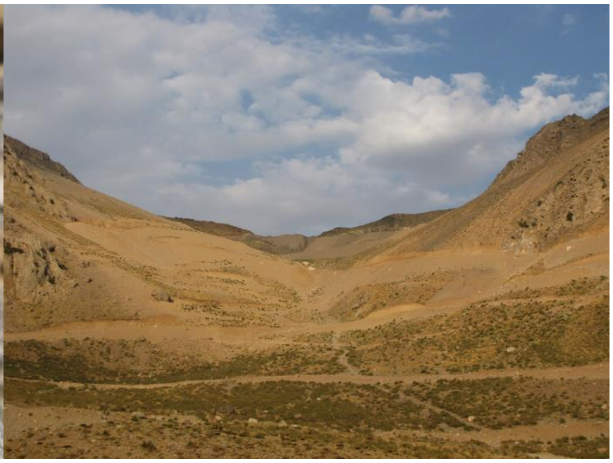

Figure S17. *Paralaudakia microlepis*. Figure S18. Habitat of *Paralaudakia microlepis*, Kohgiluyeh and Boyer-Ahmad province.

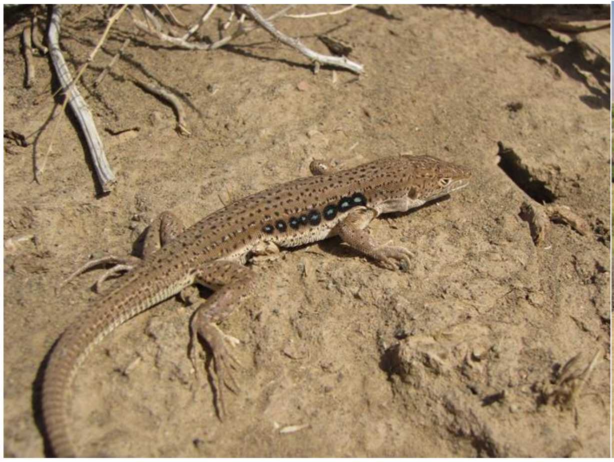

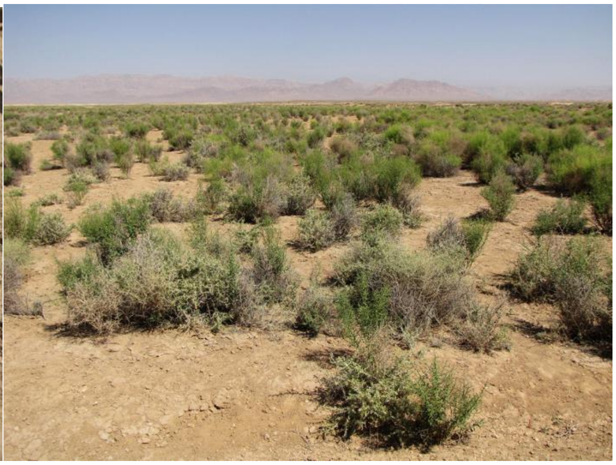

Figure S19. *Eremias velox*. Figure S20. Habitat of *Eremias velox*, Semnan province.

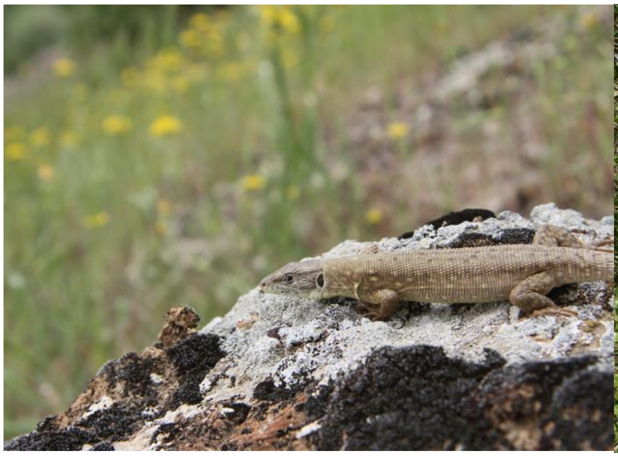

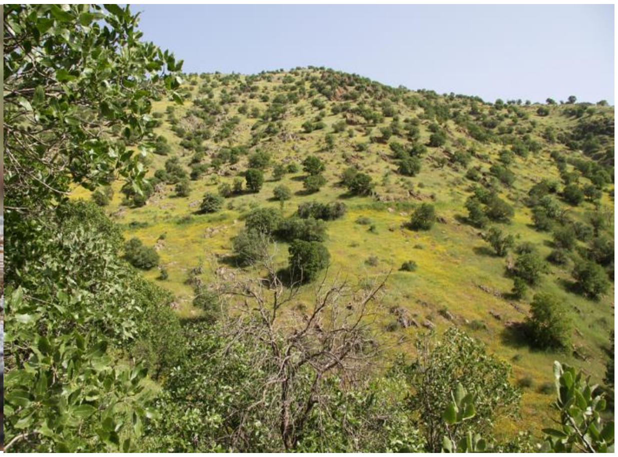

Figure S21. *Timon kurdistanicus*. Figure S22. Habitat of *Timon kurdistanicus*, Kurdistan province.

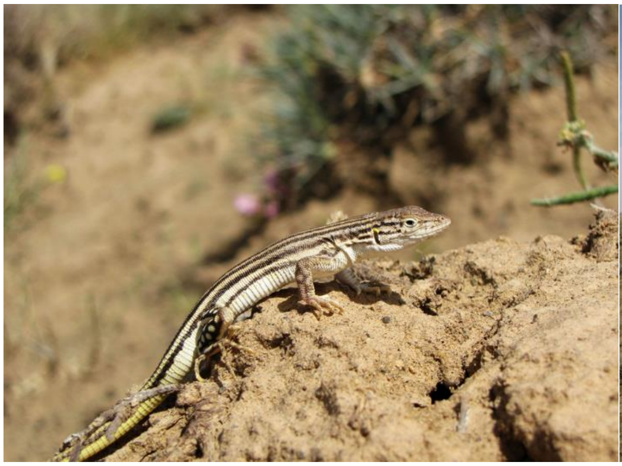

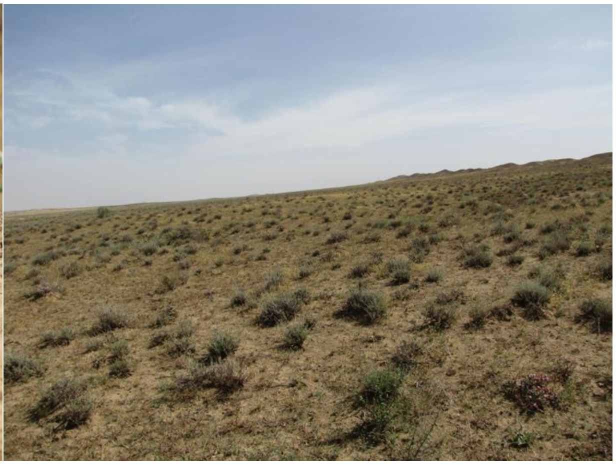

Figure S23. *Eremias fasciata*. Figure S24. Habitat of *Eremias fasciata*, Elburz province.

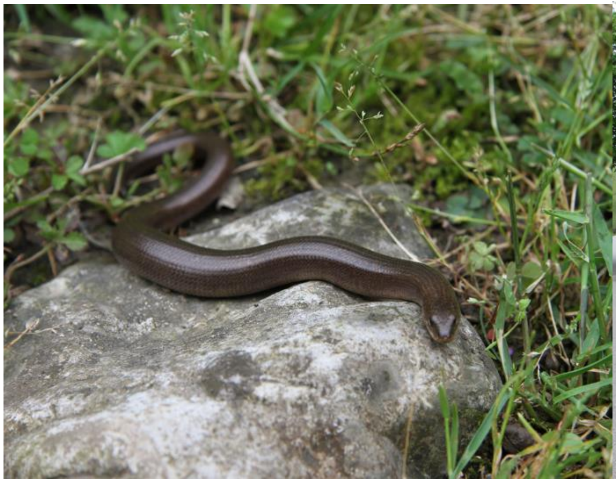

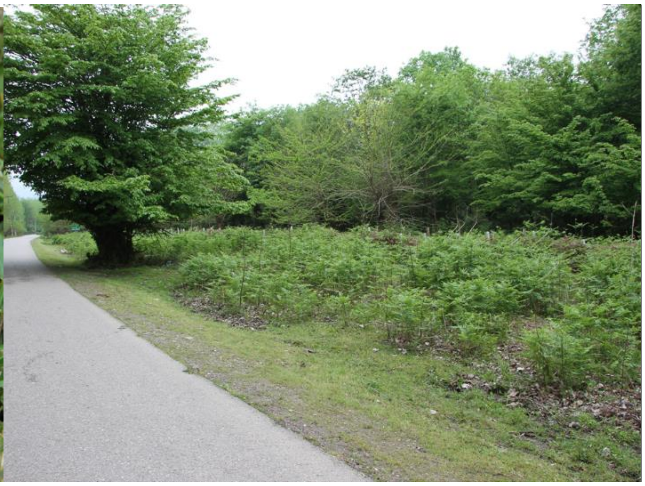

Figure S25. *Anguis colchica*. Figure S26. Habitat of *Anguis colchica*, Mazandaran province.

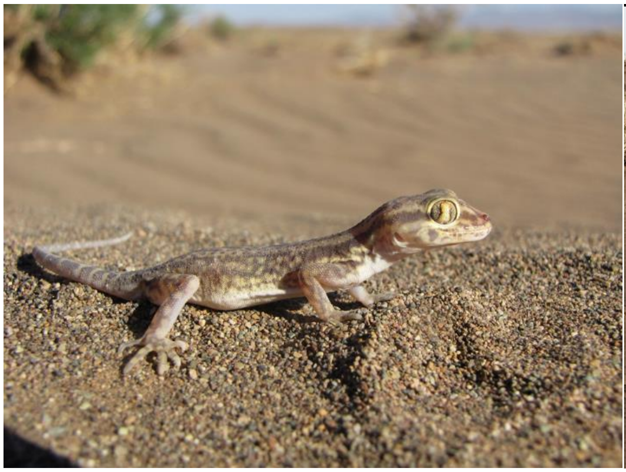

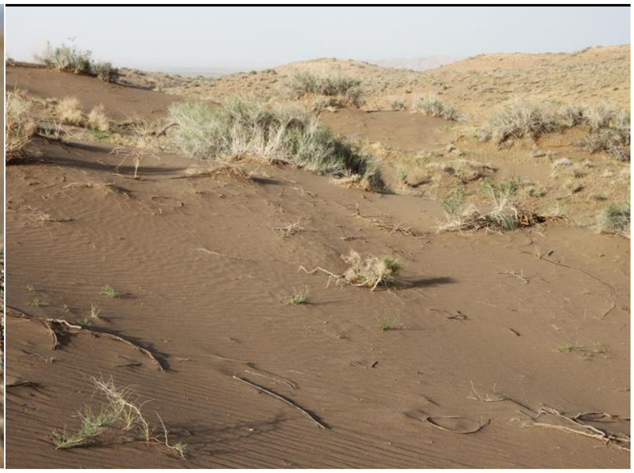

Figure S27. *Crossobamon eversmanni*. Figure S28. Habitat of *Crossobamon eversmanni*, Khorasan-e-Razavi province.

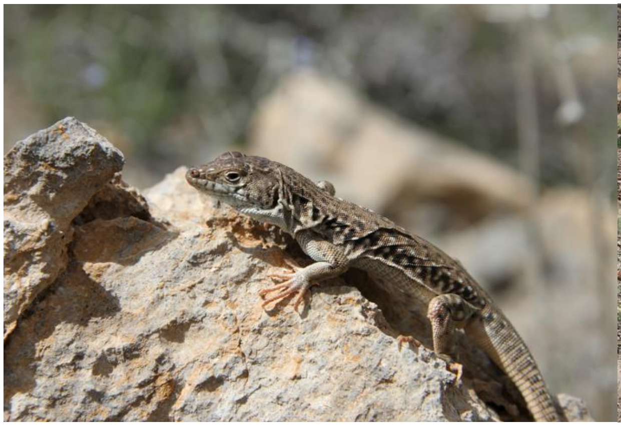

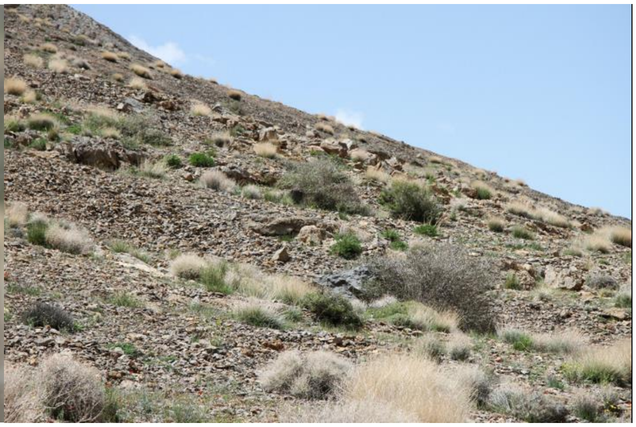

Figure S29. *Eremias isfahanica*. Figure S30. Habitat of *Eremias isfahanica*, Isfahan province.

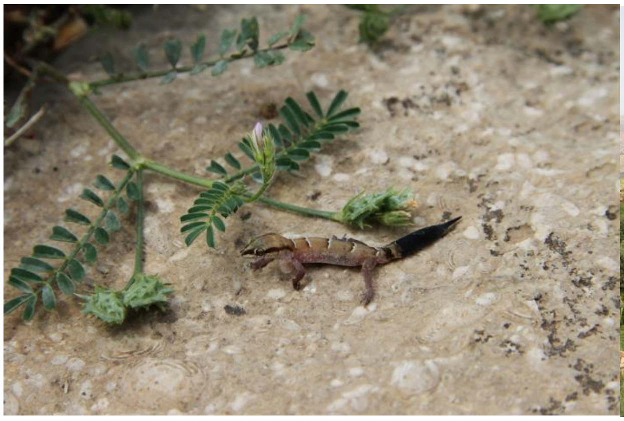

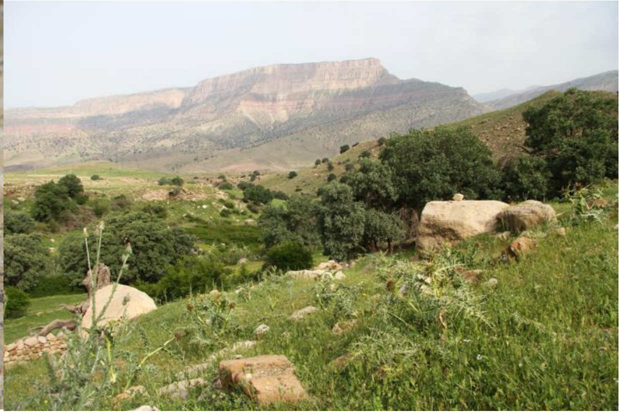

Figure S31. *Microgecko helenae*. Figure S32. Habitat of *Microgecko helenae*, Luristan province.

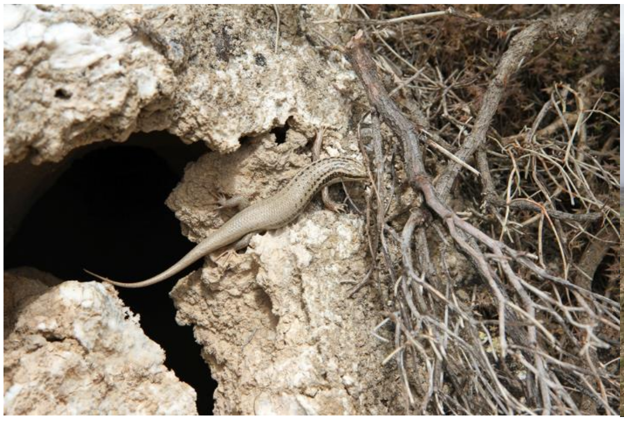

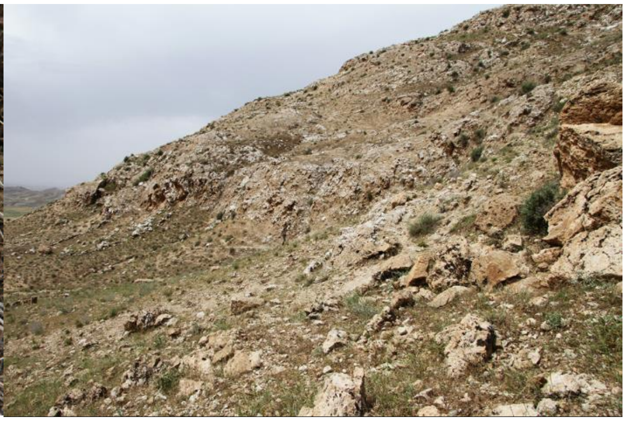

Figure S33. *Heremites septemtaeniata*. Figure S34. Habitat of *Heremites septemtaeniata*, Ilam province.

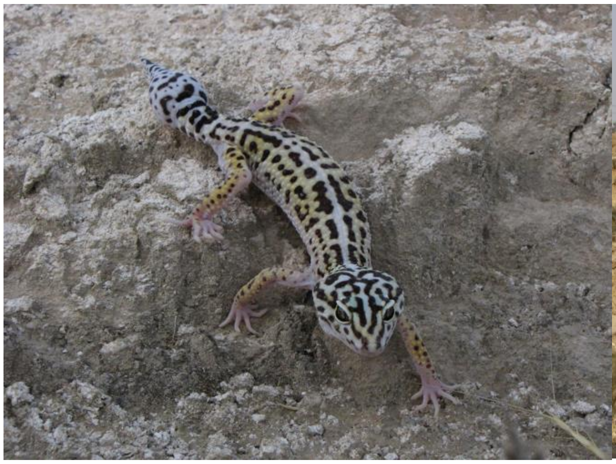

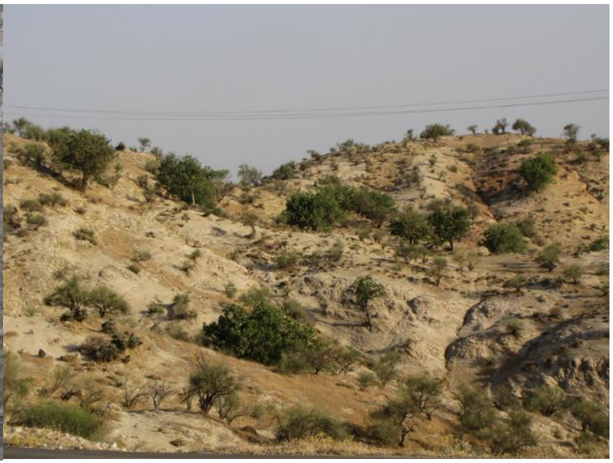

Figure S35. *Eublepharis angramainyu*. Figure S36. Habitat of *Eublepharis angramainyu*, Fars province.

Appendix S3. Papers and books examined in this study.

Akbarpour, M. *et al.* A new species of frog-eyed gecko, genus *Teratoscincus Strauch*, 1863 (Squamata: Sphaerodactylidae), from southeastern Iran. *Zool. Middle East.* **63**, 296-302 (2017).

Ahmadzadeh, F. *et al.* Cryptic speciation patterns in Iranian rock lizards uncovered by integrative taxonomy. *PLoS ONE.* **8**, e80563 (2013).

Ahmadzadeh, F., Flecks, M., Torki, F. & Böhme, W. A new species of angular-toed gecko, genus *Cyrtopodion* (Squamata: Gekkonidae), from southern Iran. *Zootaxa*. **2924**, 22-32 (2011).

Anderson, S. C. *The Lizards of Iran*. (Society for the Study of Amphibians and Reptiles, New York, 1999).

Anderson, SC. A new species of Bunopus (Reptilia: Gekkonidae) from Iran and a key to lizards of the genus *Bunopus*. *Herpetologica* **29**, 355–358 (1973).

Auer, M., Richter, S. & Khani, A. A new record of the Turkmenian fat-tailed gecko, *Eublepharis turcmenicus* Darevsky, 1978, from north-eastern Iran (Squamata: Gekkonidae). *Zool. Middle East*. **45**, 107-109 (2008).

Bahmani, Z., Rastegar-Pouyani, N. & Gharezi, A. A new record of *Eremias montanus* Rastegar-Pouyani & Rastegar-Pouyani, 2001 (Sauria: Lacertidae) from Kurdistan Province, Western Iran. *Amphib. reptile conserv*. **5**, 11-14 (2011).

Bahmani, Z., Rastegar-Pouyani, E. & Rastegar-Pouyani N. The phylogenetic relationships and molecular systematics of scincid lizards of the genus *Heremites* (Sauria, Scincidae) in the Middle East based on mtDNA sequences. *Mitochondrial DNA Part A* **29**, 846-855 (2018).

Dakhteh, S.M.H., Kami, H.G. & Anderson, S.C. *Stenodactylus khobarensis* (Haas, 1957): an addition to the Iranian herpetofauna (Reptilia: Squamata: Gekkonidae). *Russ. J. Herpetol*. **14**, 229–231 (2007).

Eskandarzadeh, N. *et al.* Annotated checklist of the endemic Tetrapoda species of Iran. *Zoosystema*. **40**, 507-537 (2018).

Fahimi, H., Papenfuss, T.J. & Anderson, S.C. Geographic distribution: Iran, Khuzestan province: *Scincus mitranus*. *Herpetol. Rev*. **40**: 454 (2009).

Faizi, H. & Rastegar-Pouyani, N. Intra- and-inter-specific geographic variation in the Iranian Scincid lizards of the genus *Trachylepis* Fitzinger 1843 (Sauria: Scincidae). *Ira. J. Anim. Biosys*. **2**, 1–11 (2006).

Faizi, H., Rastegar-Pouyani, N. & Yarani, R. On the occurrence of ectoparasite ticks on *Trachylepis* and *Eumeces* (Reptilia: Scincidae) in Iran. *Amphib. reptile conserv*. **5**, 7-10 (2011).

Faizi, H. *et al.* A new species of *Eumeces* Wiegmann 1834 (Sauria: Scincidae) from Iran. *Zootaxa*. **4320**, 289-304 (2017).

Farhadi Qomi, M., Kami, H. G., Shajii, H. & Kazemi, S. M. Further recordes of the plateau snake shink *Ophiomorus nuchalis* Nilson and Andren, 1978 (Sauria: Scincidae) from Isfahan province, Iran. Ira. *J. Anim. Biosys.* **7**, 171-175 (2011).

Fathinia, B. & Rastegar-Pouyani, N. Sexual dimorphism in *Trapelus ruderatus ruderatus* (Sauria: Agamidae) with notes on the natural history. *Amphib. reptile conserv.* **5**, 15-22 (2011).

Fathinia, B., Gholamifard, A. & Rastegar-Pouyani, N. First record of *Stenodactylus arabicus* (Haas, 1957) from Iran. *Herpetozoa*. **26**, 169-173 (2014).

Fathinia, B., Karamiani, R., Darvishnia, H., Heidari, N. & Rastegar-Pouyani, N. A new species of *Carinatogecko* (Sauria: Gekkonidae) from Ilam Province, western Iran. *Amphib. Reptile Conserv.* **5**, 61-74 (2011).

Fathinia, B. & Rastegar-Pouyani, N. Sexual dimorphism in *Trapelus ruderatus ruderatus* (Sauria: Agamidae) with notes on the natural history. *Amphib. Reptile Conserv.* **5**, 15-22 (2011).

Fathinia, B., Rastegar-Pouyani, N., Sampour, M., Bahrami, A. M. & Jaafari, G. The lizard fauna of the Ilam province, southwestern Iran. *Iran. J. Anim. Biosyst.* **5**, 65-79 (2009).

Gholamifard, A. & Rastegar-Pouyani, N. Distribution of *Hemidactylus* geckos (Reptilia: Gekkonidae) in Fars Province, Southern Iran. *Amphib. Reptile Conserv.* **5**, 1-6 (2011).

Heidari, N., Faizi, H., Rastegar-Pouyani, N. & Rastegar-Pouyani, E. A New Record of *Acanthodactylus cantoris* (Sauria: Lacertidae) and its comparison with closely related *A. blanfordi* in Southeastern Iran. *Asian Herpetol. Res.* **3**, 79-82 (2012).

Gholamifard, A., Rastegar-Pouyani, N. & Esmaeili, H.R. Annotated checklist of reptiles of Fars Province, southern Iran. *Iran. J. Anim. Biosyst.* **8**, 155-167 (2012).

Gholamifard, A. & Rastegar-Pouyani, N. *Microgecko helenae helenae* Nikolsky, 1907 (Sauria: Gekkonidae) extends its range to the southern Zagros Mountains, south of Iran. *Russ. J. Herpetol.* **22**, 1-10 (2015).

Gholamifard, A., Rastegar-Pouyani, N. & Rastegar-Pouyani, E. Rediscovery of *Microgecko helenae fasciatus* (Schmidtler and Schmidtler, 1972) from Kermanshah Province, Western Iran with notes on taxonomy, morphology, and habitat. *Asian Herpetol. Res*. **6**, 339–346 (2015).

Gholamifard, A. & Rastegar-Pouyani, N. First record and range extension of Sistan racerunner, *Eremias fasciata*, Blanford 1874, (Sauria: Lacertidae) from Hormozgan Province, southern Iran. *Amphib. Reptile Conserv.* **9**, 54–57 (2015).

Gholamifard, A., Rastegar-Pouyani, N. & Rastegar-Pouyani, E. Rediscovery of *Microgecko helenae fasciatus* (Schmidtler and Schmidtler, 1972) from Kermanshah Province, Western Iran with notes on taxonomy, morphology, and habitat. *Asian Herpetol. Res.* **6**, 339-346 (2015).

Gholamifard, A. *et al.* A new species of the genus *Microgecko Nikolsky*, 1907 (Sauria: Gekkonidae) from southern Iran. *Zootaxa* **4093**: 026-040 (2016).

Gholamifard, A., Rastegar-Pouyani, N. & Rastegar-Pouyani, E. A new species of the genus *Microgecko* Nikolsky, 1907 (Sauria: Gekkonidae) from the southern Zagros Mountains, Iran. *Zootaxa* **4648**, 435–454 (2019).

Damadi, E, Gholamifard, A. & Rastegar-Pouyani, N. Additional records for *Ophiomorus brevipes* (Blanford, 1874) and *O. tridactylus* from Sistan and Baluchestan Province, Southeastern Iran. *Ira. J. Anim. Biosys*. **11**, 173-178 (2015).

Heidari, N., Rastegar-Pouyani, N., Rastegar-Pouyani, E., & Rajabizadeh, M. A new species of *Acanthodactylus Fitzinger* 1834 (Sauria: Lacertidae) from southern Iran. *Zootaxa* **3722**: 333-346 (2013).

Hosseinian Yousefkhani, S.S, Yousefi, M., Khani, A. & Rastegar Pouyani, E. Some remarks on the distribution and habitat preferences of the *Eremias strauchi kopetdaghica* Szczerbak, 1972 (Sauria: Lacertidae from the northeastern Iranian Plateau. *Herpetol. Notes*. **6**, 97-99 (2013).

Hosseinian Yousefkhani, S.S. *et al.* Environmental suitability and distribution of the Caucasian Rock Agama, *Paralaudakia caucasia* (Sauria: Agamidae) in Western and Central Asia. *Asian Herpetol. Res.* **4**, 207-213 (2013).

Hosseinian Yousefkhani, S.S., Yousefi, M., Rastegar-Pouyani, E. & Rastegar-Pouyani, N. Lizards from Qeshm Island, Iran. *Herpetol. Rev.* **44**, 486-488 (2013).

Hosseinian Yousefkhani, S.S., Rastegar-Pouyani, E. & Rastegar-Pouyani, N. Geographic variation in *Mesalina watsonana* (Sauria: Lacertidae) along a latitudinal cline on the Iranian Plateau. *Salamandra.* **49**, 171-176 (2013).

Hosseiian Yousefkhani, SS. et al. Description of a new species of the genus *Agamura* Blanford, 1874 (Squamata: Gekkonidae) from southern Iran. *Zootaxa* **4457**, 325–331 (2018).

Hosseinzadeh, M. S., Farhadi Qomi, M. & Kazemi, S. M. Distribution of *Ophiomorus nuchalis* Nilson & Andrén, 1978: Current status of knowledge. *Herpetozoa*. **29**, 92-95 (2016).

Kamali, A., & Anderson S. C. A. New Iranian Phrynocephalus (Reptilia: Squamata: Agamidae) from the hottest place on earth and a key to the genus *Phrynocephalus* in southwestern Asia and Arabia. *Zootaxa*. **3904**, 249-260 (2015).

Kamali, K. & Dakhteh, M. *Tropiocolotes steudneri* (Steudner's dwarf gecko). *Herpetol. Rev.* **37**, 241-242 (2006).

Kamali, K. & Mozaffari, O. New data on the distribution of thick-tailed tuberculate gecko, *Bunopus crassicauda* (Sauria: Gekkonidae) in Iran. *Herpetol. Notes*. **6**, 281-283 (2013).

Kamali, K. Geographic distributoin: *Acanthodactylus micropholis* (Persian Fringe-toed Lizard). *Herpetol. Rev.* **44**, 272-273 (2013).

Kamali, K. Geographic distribution: *Mesalina brevirostris* (Blanford's Short-nosed Desert Lizard. *Herpetol. Rev.* **44**, 274 (2013).

Karamiani, R. & Rastegar-Pouyani, N. New specimens of *Eublepharis angramainyu* Anderson & Leviton, 1966 (Sauria: Eublepharidae), from south-western regions of the Iranian Plateau. *Hamadryad*. **35**, 116-121 (2010).

Karamiani, R. & Rastegar-Pouyani, N. A new record of the keel-scaled geko, *Carinatogecko aspratilis* (Anderson, 1973) (Sauria: Gekkonidae) from western Iran. *Herpetol. Rev*. **4**, 337-339 (2011).

Karamiani, R. & Rastegar-Pouyani, N. Westernmost record of Khuzestan dwarf gecko *Tropiocolotes helenae helenae* (Nikolsky, 1907) (Sauria: Gekkonidae) from Kermanshah Province, western Iran. *Russ. J. Herpetol.* **19**, 212-216 (2012).

Karamiani, R., Gholamifard, A. & Rastegar-Pouyani, N. Additional specimens and further data on Bakhtiari dwarf gecko, *Tropiocolotes persicus bakhtiari* Minton, Anderson et Anderson, 1970 from southwestern Iran. *Russ. J. Herpetol.* **20**, 271-275 (2013).

Karamiani, R., Rastegar-Pouyani, N., Rastegar-Pouyani, E., Mamaghani Shishvani, M. & Nilson, G. First record of *Ablepharus chernovi* DAREVSKY, 1953, from Iran. *Herpetozoa* **31**, 94-97 (2018).

Kazemi, S. M., Farhadi Qomi, M., Kami, H. G. & Anderson, S. C. A new species of *Ophiomorus* (Squamata: Scincidae) from Maranjab Desert, Isfahan Province, Iran, with a revised key to the genus. *Amphib. Reptile Conserv*. **5**, 23-33 (2011).

Khani, A. *et al.* New record of the gecko *Crossobamon eversmanni* (Boulenger, 1887) from Parvand Protected Area, Khorasan Razavi province, in eastern Iran. *Herpetol. Notes*. **6**, 101-102 (2013).

Krause, V., Ahmadzadeh, F., Moazeni, M., Wagner, P. & Wilms, T.M. A new species of the genus *Tropiocolotes* Peters, 1880 from western Iran (Squamata: Sauria: Gekkonidae). *Zootaxa* **3716**, 22–038 (2013).

Mozaffari, O., Ahmadzadeh, F., Saberi-Pirooz, R. Fahimi's racerunner, a new species of the genus *Eremias* Fitzinger, 1834 (Sauria: Lacertidae) from Iran. *Zootaxa* **4768**, 565-578 (2020).

Mahroo, B.S., Ghaffari, H., Panah, A.S., Fahimi, H., Naderi, A. & Bromand, S. New geographic distribution records of Zarudny's bent-toed gecko, *Mediodactylus russowii* zarudnyi Nikolsky, 1899 (Sauria: Gekkonidae) from Iran. *Russ. J. Herpetol.* **20**, 73-78 (2013).

Mobaraki, A., Kami, H. G., Abtin, E. & Dehgannejhad, M. New records of Indian Garden Lizard, *Calotes versicolor* (Daudin, 1802) from Iran (Sauria: Agamidae). *Iranian Iran. J. Anim. Biosyst.* **9**, 147-152 (2013).

Moradi, N. & Shafiei, S. New record of the Western leopard gecko, *Eublepharis angramainyu* Anderson & Leviton, 1966 (Sauria: Eublepharidae) from southeastern Iran. *Amphib. Reptile Conserv.* **5**, 88-91 (2011).

Moradi, N., Shafiei, S., Fahimi, H., & Bromand, S. Additional information on Misonne's swollen-nose gecko, *Rhinogecko misonnei* de Witte, 1973 (Squamata, Geckonidae) in Iran. *Amphib. Reptile Conserv.* **5**, 54-60 (2011).

Mozaffari, O. Geographic distribution: *Acanthodactylus cantoris*. *Herpetol. Rev.* **41**, 510-511 (2010).

Mozaffari, O., Ahmadzadeh, F., & Parham, J. F. *Eremias papenfussi* sp. nov., a new lacertid lizard (Sauria: Lacertidae) from Tehran Province, Iran. *Zootaxa*. **3114**: 57-62 (2011).

Mozaffari, O., Ghaffari, H., Kamali, K., & Safaei, B. New record of the plateau snake skink, *Ophiomorus nuchalis* (Squamata: Scincidae), from Iran. *Russ. J. Herpetol.* **18**, 36-38 (2011).

Mozaffari, O., & Parham, J. F. A new species of racerunner lizard (Lacertidae: *Eremias*) from Iran. Proc. Calif. *Acad. Sci*. **58**, 569-574 (2007).

Mozafari, O., Kamali, K., & Fahimi, H. The atlas of reptiles of Iran. Jahad Daneshgahi, Kharazmi, Karaj, Iran (2016).

Nazari-Serenjeh, F. & Torki, F. Additional specimens of the gecko *Asaccus nasrullahi* Werner, 2006 (Reptilia: Phyllodactylidae), with notes on taxonomy and ecology. *Zool. Middle East*. **44**, 57-66 (2008).

Nazarov, R. A., & Rajabizadeh, M. A new species of angular-toed gecko of the genus *Cyrtopodion* (Squamata: Sauria: Gekkonidae) from south-east Iran (Sistan- Baluchistan Province). *Russ. J. Herpetol.* **14**, 137-144 (2007).

Nazarov, R., Ananjeva, N. & Radjabizadeh, M. Two new species of angular-toed geckoes (Squamata: Gekkonidae) from south Iran. *Russ. J. Herpetol.* **16**, 311-324 (2009).

Nazarov, R., Rajabizadeh, M., Heidari, N. & Faizi, H. Geographic distribution: *Acanthodactylus boskianus* (Bosc's Fringe-toed Lizard). *Herpetol. Rev.* **42**, 567 (2011).

Nazarov, R. A. & Rajabizadeh, M. A new species of angular-toed gecko of the genus *Cyrtopodion* (Squamata: Sauria: Gekkonidae) from south-east Iran (Sistan-Baluchistan province). *Russ. J. Herpetol.* **14**, 137-144 (2007).

Nazarov, R. A. The new record of spiny-tailed thin-toed gecko *Mediodactylus spinicauda* (Strauch, 1887), (Reptilia, Gekkonidae) in East Iran. *Russ. J. Herpetol.* **12**, 124 (2005).

Nazarov, R. A., Bondarenko, D. A. & Radjabizadeh, M. A new species of thin-toed geckos *Cyrtopodion sensu lato* (Squamata: Sauria: Gekkonidae) from Hormozgan province, south Iran. *Russ. J. Herpetol.* **19**, 292-298 (2012).

Nazarov, R. A. *et al.* A New Species of Frog-Eyed Gecko, Genus *Teratoscincus Strauch,* 1863 (Squamata: Sauria: Sphaerodactylidae), from Central Iran. *Russ. J. Herpetol.* **24**, 291-310 (2017).

Nazarov, RA., Melnikov, DA., Rajabizadeh, M. & Poyarkov, NA. A new species of short-fingered geckos *Stenodactylus* (Squamata, Geckonidae) from South Iran with taxonomic notes on validity of the genus *Trigonodactylus* Hass, 1957. *Zootaxa* **4457**, 093–113 (2018).

Nilson, G. & Andrén, C. A new species of *Ophiomorus* (Sauria: Scincidae) from Kavir Desert, Iran. *Copeia* 4, 559-564 (1978).

Nilson, G., Rastegar-Pouyani, N., Rastegar-Pouyani, E. & Andrén, C. Lacertas of South and Central Zagros Mountains, Iran, with descriptions of two new taxa. *Russ J. Herpetol*. **10**, 11–24 (2003).

Oraie, H., Rahimian, H., Rastegar-Pouyani, N., Rastegar-Pouyani, E. & Khosravani, A. The easternmost record of *Ophisops elegans* (Sauria: Lacertidae) in Iran. *Herpetol. Notes* **5**, 469-470 (2012).

Parsa, H., Oraie, H., Khosravani, A. & Rastegar-Pouyani, N. Systematics and distribution of the Iranian Plateau leaf-toed geckos of the genus *Asaccus* (Sauria: Gekkonidae). *Iran. J. Anim. Biosyst.* **5**, 43-55 (2009).

Rajabizadeh, M., Faizi, H., Anderson, S. C., Zarrintab, M. & Nazarov, R. Taxonomic status of *Tropiocolotes* cf. *steudneri* with a description of a new species of *Tropiocolotes* (Reptilia: Squamata: Gekkonidae) in southern Iran. *Zootaxa*. **4388**, 283- 291 (2018).

Rajabizadeh, M., Nilson, G. & Kami, H. G. A new species of mountain viper (Ophidia: Viperidae) from the Central Zagros Mountains, Iran. *Russ. J. Herpetol*. **18**, 235-240 (2011).

Rajabizadeh, M. *et al.* New records of lacertid genera, *Iranolacerta* and *Apathya* (Sauria: Lacertidae) in Iran. *Iran. J. Anim. Biosyst.* **6**, 21-32 (2010).

Rajabizadeh, M., Ghasemi, M., Faizi, H., & Rastegar-Pouyani, N. New data on tropiocolotes cf. Steudneri (Peters, 1869), (Sauria: Gekkonidae) in southern Iran. Russ. 1827 (Reptilia: Squamata: Gekkonidae). *Sauria* **33**, 47-56 (2010).

Rajabizadeh, M., Rastegar-Pouyani, N., Faizi, H. & Bostanchi, H. New insights in the taxonomy of the Persian Sand Gecko *Tropiocolotes persicus* (Nikolsky, 1903) (Sauria: Gekkonidae). *Zool. Middle East* **49**, 55–62 (2010).

Rajabizadeh, M. & Rastegar-Pouyani, N. Two new records of reptiles (Reptilia: Squamata) from southeastern Iran. *Turk. J. Zool*. **33**, 103-104 (2009).

Rastegar-Pouyani, E., Avci, A., Kumlutas, Y., Ilgaz, Ç. & Hosseinian Yousefkhani, S. S. New country record and range extension of *Eremias suphani* Başoğlu & Hellmich, 1968 from Iran. *Amphib. Reptile Conserv*. **6**, 35-39 (2013).

Rastegar-Pouyani, E., Rastegar-Pouyani, N., Hosseinian Yousefkhani, S. S. & Arab, M. Rediscovery of *Darevskia steineri* (Eiselt, 1995) (Sauria: Lacertidae) from Iran. *Russ. J. Herpetol*. **20**, 1-3 (2013).

Rastegar-Pouyani, E. *et al.* A new species of the genus *Eremias* Fitzinger, 1834 (Squamata: Lacertidae) from Central Iran, supported by mtDNA sequences and morphology. *Zootaxa* **4132**, 207-220 (2016).

Rastegar-Pouyani, N. A new species of *Asaccus* from the Zagros Mountains, Kermenshahan province, Western Iran. *Russ. J. Herpetol*. **3**, 11–17 (1996).

Rastegar-Pouyani, N. & Nilson, G. A new species of *Eremias* from Fars province, south-Central Iran. *Russ. J. Herpetol*. **4**, 94-101 (1997).

Rastegar-Pouyani, N. & Nilson, G. A new species of *Lacerta* from the Zagros Mountains, Esfahan province, west-central Iran. *Proc. Calif. Acad. Sci.* **50**, 267-277 (1998).

Rastegar-Pouyani, N. (1998) A new species of *Acanthodactylus* (Sauria: Lacertidae) from Qasr-e-Shirin, Kermanshah Province, Western Iran. *Proc. Calif. Acad. Sci.* **50**, 257–265.

Rastegar-Pouyani, N. First record of the lacertid *Acanthodactylus boskianus* (Sauria: Lacertidae) for Iran. *Asiat. Herpetol. Res*. **8**, 85-89 (1999).

Rastegar-Pouyani, N. Two new subspecies of *Trapelus agilis* Complex (Sauria: Agamidae) From Lowland Southwestern Iran and Southeastern Pakistan. *Asiat. Herpetol. Res*. **8**, 90-101(1999).

Rastegar-Pouyani, N. & Rastegar-Pouyani, E. A new species of *Eremias* (Sauria: Lacertidae) from highlands of Kermanshah Province, western Iran. *Asiat. Herpetol. Res*. **9**, 107-112 (2001).

Rastegar-Pouyani, N. & Rastegar-Pouyani, E. A new form of *Eremias* (Sauria: Lacertidae) from the Alvand Mountains, Hamedan Province, Western Iran. *Iran. J. Anim. Biosyst.* **1**, 14-20 (2005).

Rastegar-Pouyani, N., Nilson, G. & Faizi, H. A new species of *Asaccus* (Sauria: Gekkonidae) from Kurdistan province, western Iran. *Hamadryad*. **30**, 141-150 (2006).

Rastegar-Pouyani, N., Rastegar-Pouyani, E. & Jawaheri, M. *Field guide to the reptiles of Iran*. (Razi University Press, Razi, 2007).

Rastegar-Pouyani, N., Oraei, H. & Johari, M. New records of the geckonid lizard, *Cyrtopodion heterocercum heterocercum* (Sauria: Gekkonidae) from Isfahan Province, Central Iran, with an extended descritpion and notes on distribution. *Russ. J. Herpetol*. **16**, 220-228 (2009).

Rastegar-Pouyani, N., Oraei, H. & Johari, M. A new record of the gekkonid lizard *Tropiocolotes latifi* Leviton & Anderson, 1972 from Iran (Sauria: Gekkonidae). *Zool. Middle East*. **47**, 105-107 (2009).

Rastegar-Pouyani, N., Khosravani, A. & Oraie, H. A new record of *Cyrtopodion scabrum* (Heyden, 1827) from the Caspian Sea coastal region, Guilan Province, northern Iran. *Herpetol. Notes*. **3**, 61-63 (2010).

Rastegar-Pouyani, N., Karamiani, R., Oraei, H., Khosrawani, A. & Rastegar-Pouyani, E. A new subspecies of *Darevskia raddei* (Boettger, 1892) (Sauria: Lacertidae) from the West Azerbaijan Province, Iran. *Asian Herpetol. Res.* **2**, 216-222 (2011).

Safaei, B. et al. New geographic distribution records of zarudny's bent–toed gecko, *Mediodactylus russowii zarudnyi* Nikolsky, (1899) (Sauria: Gekkonidae) from Iran. *Russ. J. Herpetol*. **201**, 73 – 78 (2012).

Safaei-Mahroo, B. *et al.* The herpetofauna of Iran: checklist of taxonomy, distribution and conservation status. *Asian Herpetol. Res.* **6**, 257-290 (2015).

Safaei-Mahroo, B. *et al*. A new genus and species of gekkonid lizard (Squamata: Gekkota: Gekkonidae) from Hormozgan Province with a revised key to gekkonid genera of Iran. *Zootaxa* **4109**: 428-444 (2016).

Sami, S., Safaei-Mahroo, B. & Ghaffari, H. Range extensions of three endemic snake-skinks (Scincidae: *Ophiomorus*) in Iran. *Russ. J. Herpetol*. **24**, 329 – 332 (2017).

Shafiei, S., Sehhatisabet, M.E. & Moradi, N New record of Sandfish Skink, *Scincus scincus conirostris* Blanford, 1881 (Sauria: Scincidae) from southeastern Iran. *Check List* **11**, 1774 (2015).

Šmíd J., *et al.* Annotated checklist and distribution of the lizards of Iran. *Zootaxa* **3855**: 1-97 (2014).

Šmíd, J. & Frynta, D. Genetic variability of *Mesalina watsonana* (Reptilia: Lacertidae) on the Iranian plateau and its phylogenetic and biogeographic affinities as inferred from mtDNA sequences. *Acta Herpetol.* **7**, 139-153 (2012).

Torki, F. Sexual dimorphism in the Banded Dwarf Gecko, *Tropiocolotes helenae fasciatus* (Gekkonidae) on the western Iranian Plateau. *Zool. Middle East*. **40**, 33–38 (2007).

Torki, F. et al. Geckos of the genera *Tropiocolotes* and *Asaccus* in the Zagros Mountains, Iran. *Gekko* **5**, 31–43 (2008).

Torki, F. Sexual dimorphism of scale keeling in *Asaccus kurdistanensis* Rastegar-Pouyani, Nilson & Faizi 2006. *Herpetozoa* **22**, 79-82 (2009).

Torki, F. Distribution, lifestyle, and behavioral aspects of the Iranian Fat-tailed Gecko, *Eublepharis angramainyu* Anderson and Leviton, 1966. *Gekko* **6**, 17-22 (2010).

Torki, F. Notes on sexual size dimorphism in the Iranian Short-fingered Gecko *Stenodactylus affinis* (Reptilia, Gekkonidae). *Herpetol. Bull*. **113**, 30-33 (2010).

Torki, F. Description of a new species of *Carinatogecko* (Squamata: Gekkonidae) from Iran. *Salamandra* **47**, 103-111 (2011).

Torki, F., Ahmadzadeh, F., Ilgaz, Ç., Avcı, A. & Kumlutaş, Y. Description of four new *Asaccus* Dixon and Anderson, 1973 (Reptilia: Phyllodactylidae) from Iran and Turkey. *Amphib. Reptil*. **32**, 185-202 (2011).

Torki, F., Fathinia, B., Rostami, H. A. & Nazari-Serenjeh, F. Beschreibung eines neuen *Asaccus* (Sauria: Phyllodactylidae) aus dem Iran. *Sauria* **33**, 51-62 (2011).

Torki, F., Manthey, U. & Barts, M. A new *Hemidactylus* from Lorestan Province, western Iran, with notes on *Hemidactylus robustus* Heyden, 1827 (Reptilia: Squamata: Gekkonidae). *Sauria* **33**, 47-56 (2011).

Torki, F., Manthey, U., Barts, M. Ein neuer *Hemidactylus* Gray, 1825 aus der Provinz Lorestan, West- Iran, mit Anmerkungen zu *Hemidactylus robustus*. *Heyden, 1827 (Reptilia: Squamata: Gekkonidae). Sauria*, **33,** 47-56. (2011).

Yousefi, M., Khani, A., Eslahi, H. & Hosseinian Yousefkhani, S.S. Easternmost record of *Darevskia defilippii* (Camerano, 1877) from Qarchaqe Protected Area, Kopet Dagh Mountains, northeastern Iran. Available from: http:// www.lacerta.de/AS/Bibliografie/BIB_7401.pdf (Accessed 25 April 2017) (2013).

Yousefi, M., Khani, A., Shaykhi Ilanloo, S. & Rastegar Pouyani, E. Lizard's fauna of the Sabzevar with particular emphasis on the syntopic lizard and presentation of a framework for reptile distribution of Iran. *Taxon. System.* **5**, 1-16 (2013).

Yousefi, M., Khani, A., Shaykhi Ilanloo, S., Kafash, A., & Rastegar Pouyani, E. Reptile fauna of the Khajeh protected area, with assessing its similarities with physiogeographical area of the Iranian Lizards. *Taxon. System.* **22**, 13-22 (2016).

Yousefi, M., Ataei, F., Kafash, A. & Rezaei, H. The lizard fauna of Alborz Province: distribution and conservation. *Exp. Anim. Biol*. 6, 117-126 (2018).

Copy protected with Online-PDF-No-Copy.com
